# *SignatureFinder* enables sequence mining to identify cobalamin‐dependent photoreceptor proteins

**DOI:** 10.1111/febs.17377

**Published:** 2024-12-24

**Authors:** Yuqi Yu, Laura N. Jeffreys, Harshwardhan Poddar, Adam Hill, Linus Johannissen, Fanzhuo Dai, Michiyo Sakuma, David Leys, Derren J. Heyes, Shaowei Zhang, Nigel S. Scrutton

**Affiliations:** ^1^ Department of Chemistry The University of Manchester, Manchester Institute of Biotechnology UK; ^2^ Department of Chemistry The University of Manchester UK; ^3^ Present address: Astra Zeneca Cambridge UK; ^4^ Present address: Department of Biology and Chemistry, College of Sciences National University of Defense Technology Changsha China

**Keywords:** bioinformatics, cobalamin, photoreceptors, sequence motif, structure

## Abstract

Photoreceptors control cellular processes in response to light. Most photoreceptors sense blue or red light, but the recent discovery of the cobalamin‐dependent photoreceptor, CarH, has expanded the wavelength range of photoreception to other regions of the electromagnetic spectrum to include the green light region. Further identification of cobalamin‐dependent green light‐sensitive photoreceptors has been hampered owing to poor annotation of the light responsiveness of cobalamin‐binding domains (CBDs) in public databases. Here we report a computational workflow, *SignatureFinder*, that uses a combination of sequence and structural analyses to identify new light‐responsive CBD‐containing proteins. The light response of exemplar proteins containing the proposed signature were confirmed experimentally. A structural analysis of these new photoreceptors, including the crystal structure of a new CBD domain, highlights how the signature elements interact with the cobalamin chromophore to sense light. Database mining of 128 000 CBD‐containing sequences using the identified signature revealed more diverse CBD‐containing photoreceptors, thereby expanding the family of green‐light photoreceptors. A *SignatureFinder* web server is available (https://enzymeevolver.com) for wider applications, including the identification of signature sequences of other biological ligands of interest.

## Graphical abstract

The authors show the utility of their computational tool, available at https://enzymeevolver.com, for the identification of novel photoreceptors. Signature residues for the binding of the green‐light‐sensitive cofactor adenosylcobalamin were determined. The light‐sensitive reactions of exemplar proteins containing the proposed signature were confirmed experimentally through biochemical, spectroscopic and crystallographic techniques. Additional green‐ and red‐light‐induced photoreceptors are proposed.

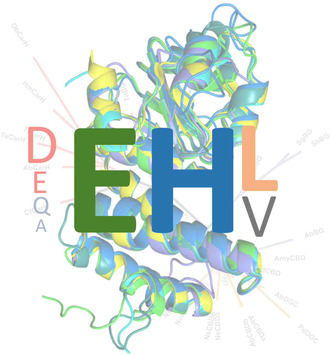

**Abbreviations**
AIC: Akaike information criterion
ANTAR: AmiR and NasR transcription antitermination regulators
BBD: biliverdin‐binding domain
Cbl: cobalamin
CBD: cobalamin‐binding domain
DBD: DNA‐binding domain
DGC: diguanylate cyclase
DICT: diguanylate cyclases and phosphodiesterases and two‐component systems
GAF: cGMP‐specific phosphodiesterases, adenylyl cyclases and FhlA
HTH: Helix‐Turn‐Helix
MEDS: MEthanogen/methylotroph DcmR Sensory
ML: maximum likelihood
MS: mass spectrometry
PDB: protein data bank
PDE: phosphodiesterase
RMSD: root‐mean‐square deviation
SEC‐MALS: size exclusion multi‐angular light scattering
SSN: sequence similarity network

## Introduction

In cell biology, optogenetics empowers researchers to use light to perturb and control cellular processes with fine spatial and temporal resolution [1, 2, 3, 4, 5]. Optogenetics has also provided valuable insights into cell malfunction for the development and improvement of therapeutics [2, 6, 7, 8]. Photoreceptors are at the core of optogenetics where they play important roles in maintaining cellular activities in response to light [9, 10, 11, 12, 13]. Photoreceptors typically comprise sensor domains and coupled effector domains [1, 14, 15]. They sense light using a bound chromophore located in the sensor domain and transmit this signal to the coupled effector domains, which undergo conformational changes to trigger a downstream response [6, 16, 17, 18, 19, 20, 21]. Photoreceptors are highly diverse in that different sensor domains combine with a range of available effector domains. Additionally, artificial photoreceptor fusion proteins can combine sensors and targeted effector domains to expand this diversity, which forms the basis of a multifunctional optogenetics toolbox [1].

CarH was the first discovered photoreceptor to use adenosylcobalamin as a light‐sensing chromophore [20]. This interesting protein regulates the expression of the genes required for the synthesis of carotenoids, which can protect cells from photo‐oxidative stress [20, 22, 23]. In the dark, CarH is a tetramer that binds to target DNA to inhibit transcription. Upon illumination with green light, adenosylcobalamin photochemistry leads to dissociation of the tetramer, which causes the release of the DNA and initiation of transcription. As a rare example of a green‐light‐induced photoreceptor, CarH has been exploited in light‐dependent cell release/recovery and regulation of cell adhesion [24, 25, 26, 27]. Future applications of CarH in establishing cell or mini‐organ culturing platforms, and optical therapeutic treatments, are also envisioned [28]. The use of CarH in medical hydrogels is limited by the permeability of green light through the skin barrier. Therefore, cobalamin‐dependent photoreceptors sensitive to other wavelengths of light may provide for further optogenetic tools, particularly proteins sensitive to red light [29]. To assist in this goal, reliable identification of new cobalamin‐binding photoreceptors is needed. A major challenge is that most cobalamin‐binding domains (CBDs) are not naturally light‐responsive, making it difficult to identify new photoreceptor proteins. For example, CBD‐containing enzymes, such as methylcobalamin‐dependent methionine synthase, adenosylcobalamin‐dependent enzymes glutamate mutase and methylmalonyl‐CoA mutase, utilise cobalamin as a nonphotoactive cofactor. As such, these thermally activated enzymes do not show the typical CarH‐like cobalamin photochemical response [30, 31, 32, 33, 34, 35]. There are 128 000 sequences containing CBDs in the protein database (InterPro [36] classification number: IPR006158), and there are no annotations for the light‐responsive features of these proteins. Methods are now required to identify signature sequences for light‐responsive CBD‐containing proteins to distinguish potential light‐responsive sequences from the thousands of other CBD‐containing proteins in the database to enable identification and experimental characterisation of cobalamin‐dependent photoreceptor proteins. We address this need here.

## Results and discussion

### Overview of approach

We set out to combine phylogenetic analysis and structural predictions to classify photoreceptors and nonphotoreceptors. Our hypothesis is that proteins similar to CarH at both the evolutionary and structural level have a strong likelihood of being photoreceptors, which was then validated by experimental analysis. First, we identified signature sequences using sequence alignments and performed biochemical analysis of potential photoreceptor‐AdoCbl models, as well as determining the crystal structure and biophysical/chemical analysis of the predicted photoreceptor *Ct*MerR to confirm light‐responsivity. Subsequent database mining based on the identified signature sequence was then used to expand the photoreceptor family to 1500 potential light‐responsive CBD‐containing photoreceptors of wide‐ranging function. A sequence similarity network (SSN) was constructed and published on NDEx [37, 38, 39] with an interactive interface to reveal the protein clusters, and representatives from each cluster were modelled by AlphaFold for annotation of functional domains. Our computational workflow is available as the web server *SignatureFinder* alongside our previous tool IREDFisher at https://enzymeevolver.com which will allow community use to explore signature sequences in protein families of interest [40]. This work also led to the characterisation of proteins that contain two chromophores allowing this photocobilin protein family to sense light across the UV–Vis spectrum [29].

### SignatureFinder web server

**Fig. 1:**
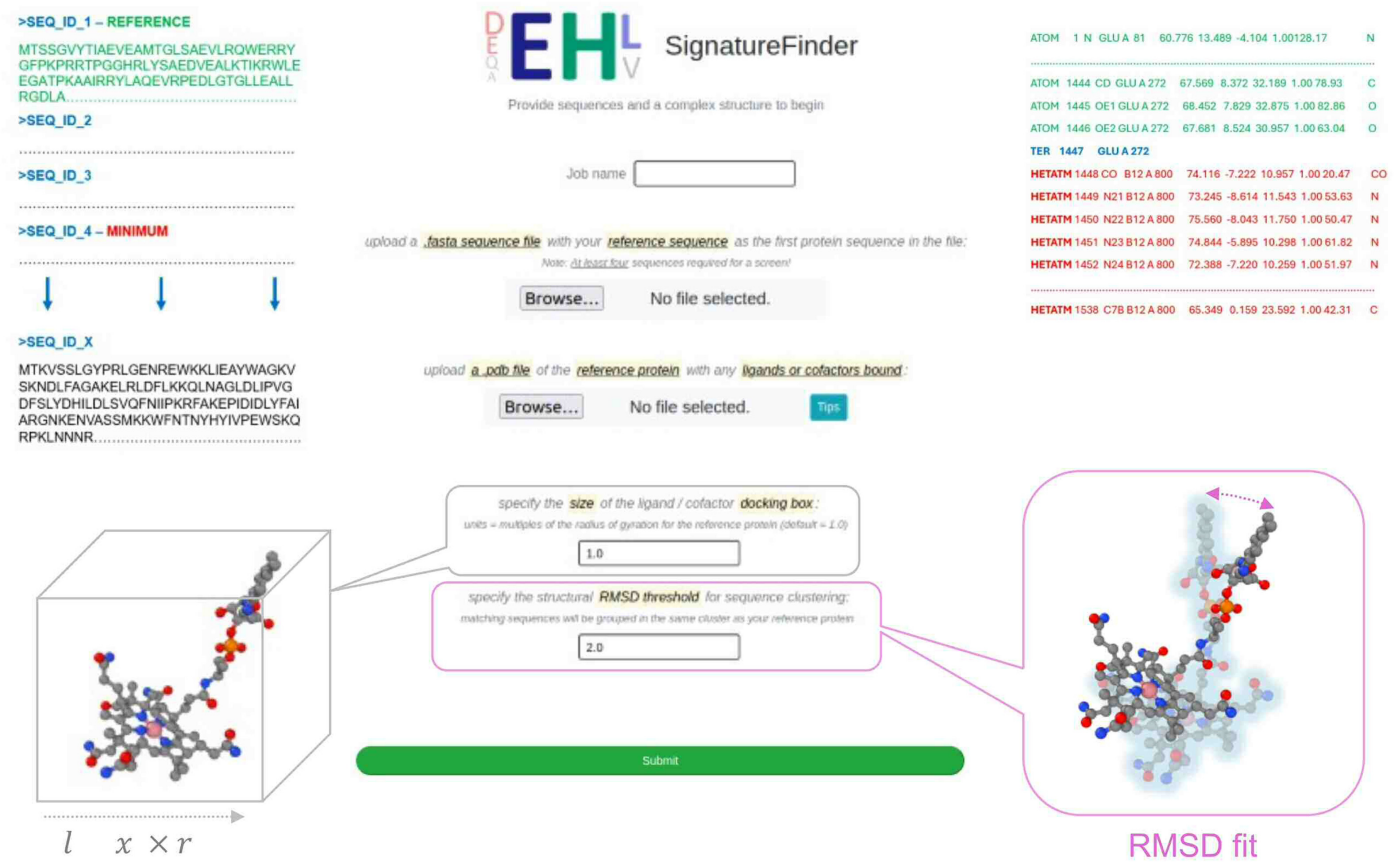
Input and output views of the *SignatureFinder* online tool. A screenshot of the *SignatureFinder* web interface showing the information required by the user. Additional information has been added to assist users in determining the data required for each section. Firstly, at least four sequences are required for the web server to begin in .fasta format. The first of these sequences must be the reference sequence for comparison. Secondly, the same reference structure should be uploaded with any cofactors bound in .pdb format. Thirdly, the size of the docking area for the cofactor should be inputted in units of multiples of the gyration radius of the reference protein. Finally, a root‐mean‐square deviation (RMSD) threshold should also be input to classify test sequences as sufficiently similar to the reference protein, and then, the query can be submitted. The web server will then output useful files including: phylogenetic tree data; pre‐ and postdocking homology models and summary files for ligands and sequences with the best RMSD scores.

We demonstrate the application of the *SignatureFinder* web server by using it to identify light‐responsive signatures in CBD‐containing proteins (Fig. 1). A reference protein known to have the function of interest is needed prior to use of the web server. The web server requires the upload of a document containing the reference FASTA sequence and at least three query FASTA sequences. A ligand‐bound structure of a reference protein is also needed for structural comparison with predicted models of the homologues. Choosing an appropriate reference protein sequence and structure are key to the success of this method and web server. In this study, CarH from *Thermus thermophilus* (*Tt*CarH) was the reference protein and CBD‐containing sequences collected by a BLAST [41] homology search based on the CBD in *Tt*CarH (*Tt*CBD) were the query sequences. The crystal structure of the CBD with a bound adenosylcobalamin molecule (PDB code: 5C8A) was used as the reference structure. To ensure that the models don't diverge from the reference proteins, a 2.0 Å cut‐off was applied to the root‐mean‐square deviation (RMSD) between the models and reference. The size of the docking box was calculated based on gyration radius of the ligand (Rg) in the reference structure. It is a cubic box with Rg * Rg * Rg. The user can specify the size by filling out the ratio from the web server. A ratio of 1.0 was used in this case and can be used as a default by users.

**Fig. 2:**
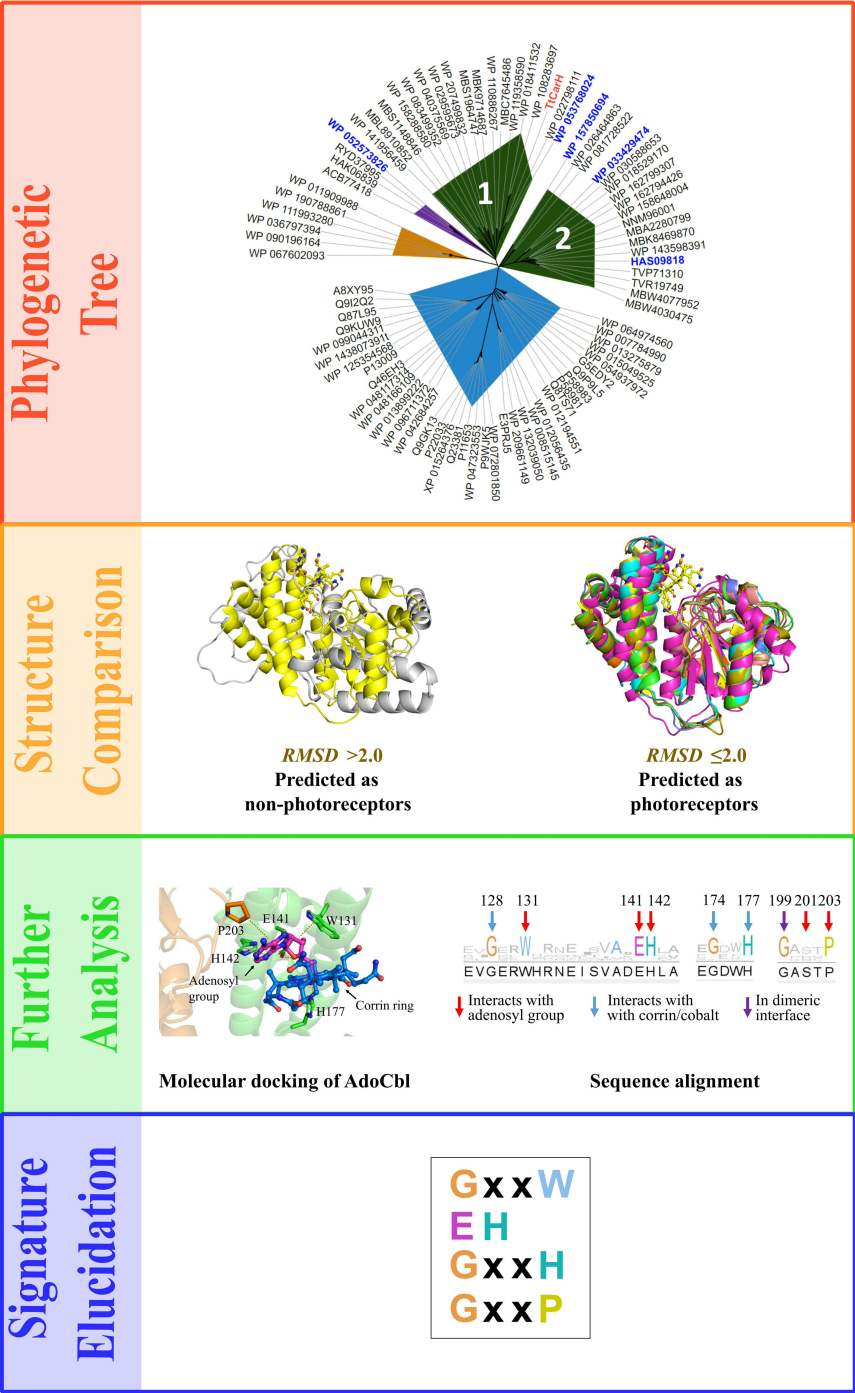
Workflow for the *SignatureFinder* online tool and further analysis. After submission of the FASTA sequences, a phylogenetic tree is generated comparing the unknown proteins to collected cobalamin‐binding domains (CBDs) using the interactive tree of life (ITOL) [41]. CBDs that structurally resemble *Tt*CarH, light‐independent enzymes and the light‐sensitive flavoprotein AppA (chosen due to its lack of cobalamin cofactor) are coloured in dark green, and orange, respectively. The two distinct groups of sequences resembling CarH in structure are labelled 1 and 2 accordingly. Group 1 contains *Tt*CarH whereas group 2 does not, suggesting interesting changes. Proteins predicted to be light insensitive are shown in blue such as methionine synthase. Sequences not modelled are coloured in purple. Each sequence is labelled by using the code from the NCBI database. For further details, see Table S1. The sequences for further experimental validation were labelled in blue font. The known photoreceptor *Tt*CarH is highlighted in red font. At the same time, structural alignments are generated. The left model resembles *Tt*CarH (PDB code: 5C8A) with RMSD <2 Å whereas the right model (WP_090196164) resembles flavoprotein AppA (PDB code: 4HH0) with RMSD = 13.1 Å (Table S1). Further analysis was conducted on the structures found to be genetically and structurally similar to CarH. The conserved interaction pattern between AdoCbl and putative light‐responsive cobalamin‐binding domains was determined using molecular docking allowing for the comparison of conservation of the signature sequences between putative light‐responsive CBDs and other CBDs. Sequences were annotated using *Tt*CarH as a reference.

The query sequences go through the workflow in a stepwise manner (Fig. 2): in the first step, a phylogenetic tree file is generated to give a general picture of the evolutionary relationship between the query sequences and the reference protein. Sequences in the same branch of the phylogenetic tree are defined as photoreceptors. Next, the 3D structure of each sequence is modelled and compared with the reference structure. Sequences where the RMSD of the model is under the cut‐off values are defined as photoreceptors and vice versa. Then, sequences of predicted photoreceptors are aligned and the light‐sensor adenosylcobalamin (AdoCbl) is docked into the structure of each predicted photoreceptor. The amino acid residues that interact with AdoCbl and are also conserved in the sequence alignment are defined as the signature for light response.

### Identification of signatures for light response in cobalamin‐binding proteins by *SignatureFinder* workflow

The phylogenetic tree file generated by *SignatureFinder* was visualised by ITOL [42] (Fig. 2) to show the evolutionary relationship between *Tt*CBD and input sequences. The input sequences show diversity by nesting in different branches of the phylogenetic tree, although they are all homologous CBDs. Sequences in blue, orange and purple areas are distant from *Tt*CarH in both evolution and structure (for structural annotation, see Table S1): generally, structures in blue resemble CBD‐dependent enzymes; structures in orange resemble flavoprotein AppA; sequences in purple were not modelled because sequence identity to the template protein was lower than 20%. AppA is an interesting photoreceptor first discovered in *Rhodobacter sphaeroides* which contains a BLUF domain that uses FAD to sense blue light [43]. This protein is therefore a good contrasting protein to use against *Tt*CarH as it uses different cofactors and is structurally very different. There are two groups of sequences resembling CarH in structure (dark green area). Group 1, which contains *Tt*CarH, is presumably from the MerR family, like *Tt*CarH itself. Intriguingly, Group 2 shows a difference in evolution but structural resemblance to *Tt*CarH. The two groups are both defined to be putative light‐responsive CBDs by *SignatureFinder*. The AdoCbl was docked into the binding site of each putative CBD model. Sequences were then aligned, and conservation of each site was viewed by Jalview [44] (Fig. 2). By comparison with other decoy CBDs, there are several conserved motifs that have been identified between residues 128 and 177 (GxxW, EH, GxxH, GxxxP), which interact with different regions of the AdoCbl cofactor in light‐responsive CBD‐containing proteins. Among them, W131, E141, H142 and H177 have been proven to be key residues in binding AdoCbl in CarH [20]. It is noteworthy that not only sequences from the MerR family, but also sequences with ambiguous annotations, such as ‘hypothetical protein’, were predicted to be light‐responsive by *SignatureFinder*. We reason that these putative unknown sequences could be new photoreceptors.

### Structural modelling and experimental validation of new CBD‐containing photoreceptors

**Fig. 3:**
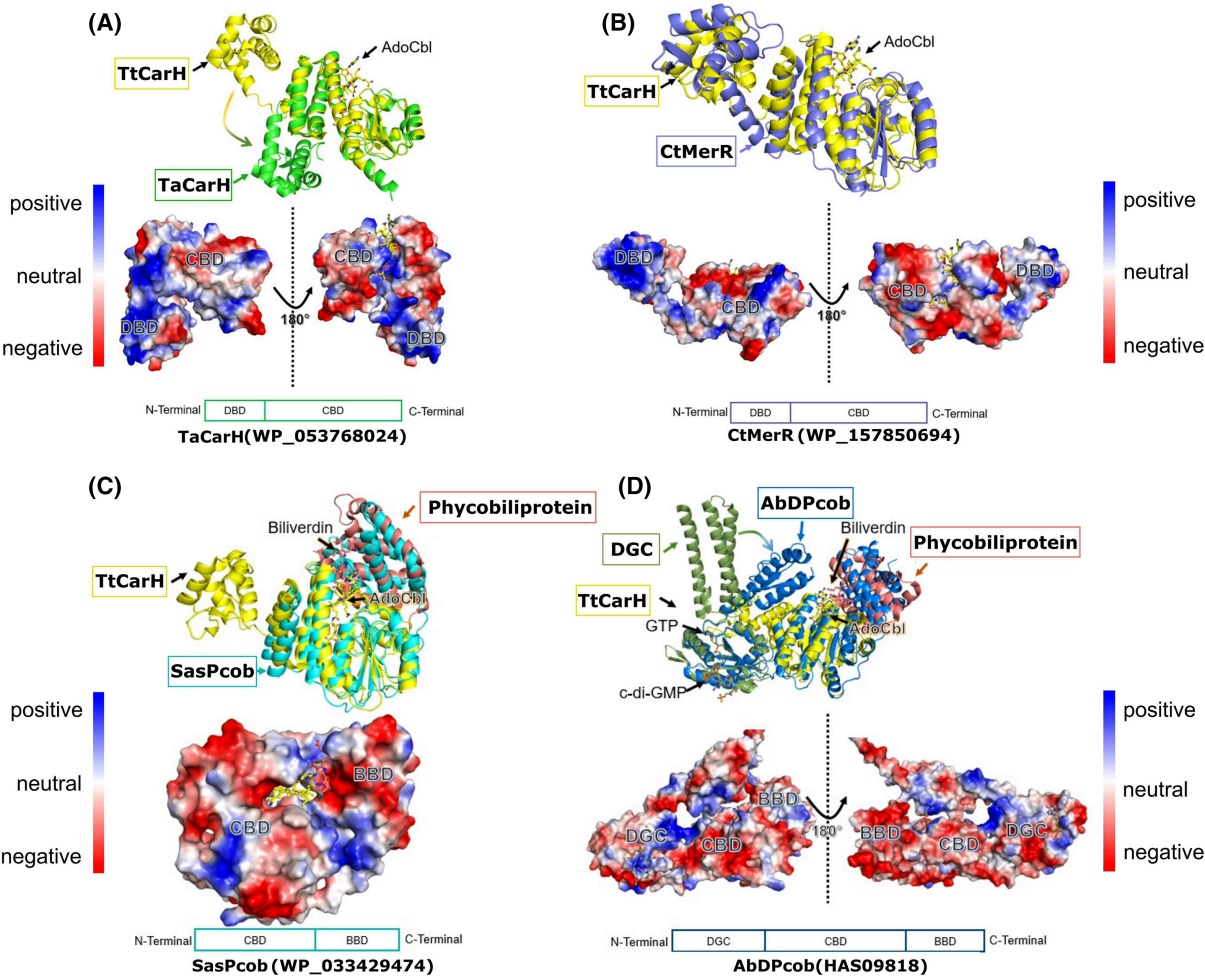
Structures of selected proteins by AlphaFold and the charge distributions on their surfaces. In the upper portion of each panel, an alignment of the protein of interest with a relevant comparison protein(s) is shown. In all cases, either *Tt*CarH (PDB: 5C8E), the phycobiliprotein SMURFP (PDB: 6FZN) and/or the diguanylate cyclase protein DgcZ (PDB: 4H54) structure is used. Proteins are labelled in coloured boxes whereas molecules and cofactors are labelled without boxes. In the lower portion of each panel, the electrostatic potential surface for each protein of interest is shown with a schematic of the protein domains and the accession code. (A) The structure of *Ta*CarH in green aligned with *Tt*CarH in yellow. The DNA‐binding domain of *Ta*CarH shows different orientation in AlphaFold models compared to *Tt*CarH. (B) The structure of *Tt*CarH (yellow) aligned with *Ct*MerR (purple). (C) The structure of *Sas*Pcob (cyan) aligned with *Tt*CarH in yellow and a phycobiliprotein in pink. (D) The structure of *Ab*DPcob in blue aligned with *Tt*CarH (yellow), a phycobiliprotein (pink) and a diguanylate cyclase (green). Structural comparisons are produced using Visual Molecular Dynamics (VMD). Accession codes are provided in Table S1.

Five sequences were selected for experimental validation (marked in blue font in Fig. 2): 1 sequence from group 1 which shares the same branch with *Tt*CarH, 3 new sequences from group 2 which are ambiguously annotated in public databases and 1 sequence from the branch close to group 1 which does not contain the light‐responsive signature as a negative control. All 5 genes were synthesised and expressed in *E. coli* (Table S1). Full‐length structures of the 5 proteins were modelled by AlphaFold [45] to view the functional domains along with CBD sensor domains (Fig. 3).

**Fig. 4:**
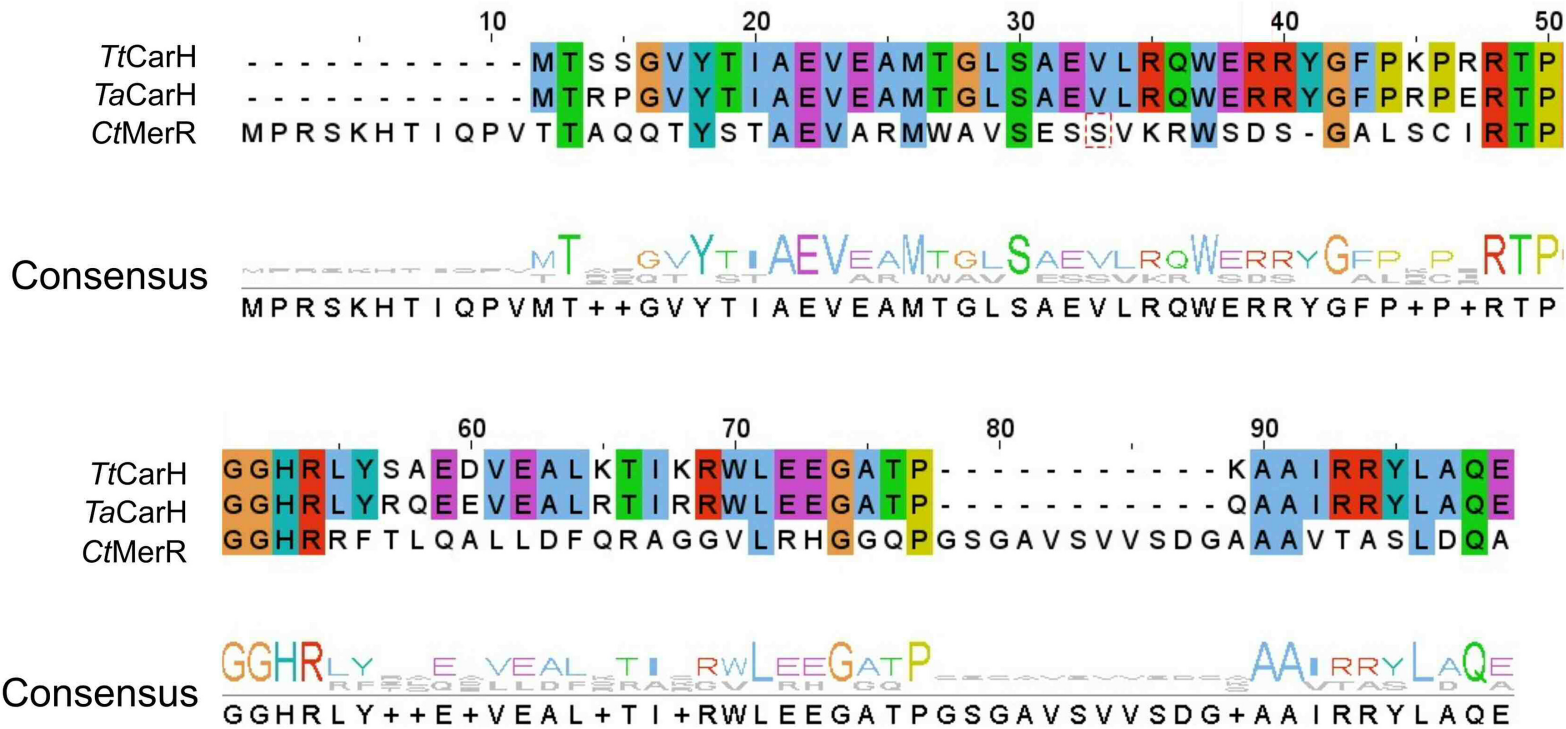
Sequence alignment in the DNA‐binding domain of *Tt*CarH, *Ta*CarH and *Ct*MerR. Shared amino acids are labelled by colour. Numbering corresponds to the *Tt*CarH sequence. Alignments were made using the MUSCLE tool from EMBL‐EBI.

Both *Ta*CarH (WP_053768024) and *Ct*MerR (WP_157850694) show positively charged N‐terminal domains for binding DNA (Fig. 3A,B), indicating they are likely transcription regulators from the MerR family. *Ta*CarH is presumably a CarH protein as it shares high sequence identity with *Tt*CarH (75% for full length and 87% for DNA‐binding domain). However, *Ct*MerR shows obvious variance in sequence particularly in the DNA‐binding domain with 32% sequence identity for full‐length protein and 25% for the DNA‐binding domain (Fig. 4). There are also significant structural differences to CarH (full‐length RMSD = 5.8 Å), suggesting that *Ct*MerR is a new light‐responsive transcriptional regulator with an alternative DNA‐binding site. For *Sas*Pcob (WP_033429474), there is a predicted biliverdin‐binding region (BBD) on the C‐terminal side of the CBD (Fig. 3C) and in the case of *Ab*DPcob (HAS09818) a diguanylate cyclase (DGC) domain was predicted (Fig. 3D), followed by a CBD and a BBD. We characterised these two proteins probing their cofactor binding and structural changes in response to light in our recent manuscript [29]. These two proteins found using *SignatureFinder* are members of the newly identified Photocobilin family. Using the biliverdin cofactor, these proteins can activate the adenosylcobalamin photochemistry observed in *Tt*CarH even using red light. The identification of these proteins highlights the ability of *SignatureFinder* to identify light‐sensitive proteins.

The full‐length structures of *Ct*MerR, *Sas*Pcob and *Ab*DPcob suggest they are new light‐induced photoreceptors, although they are all ambiguously annotated in the public database (Fig. 3; Table S1). On the other hand, WP_052573826 (*Ha*AI‐2E), which does not contain the light‐responsive signature showed a quorum‐sensing signal autoinducer‐2 exporter (AI‐2E) domain, CBD domain and a DGC domain in the AlphaFold model (Fig. S1). The CBD of *Ha*AI‐2E has limited space for accommodating the AdoCbl in comparison with *Tt*CarH as there is an arginine in the equivalent position of the conserved H177 residue that ligates to the cobalt. The light responsiveness of the CBD is not indicated in the annotation from the public database, and it is not predicted to be a photoreceptor by *SignatureFinder*.

### Biophysical analysis of *Signaturefinder*‐identified photoreceptor proteins

**Fig. 5:**
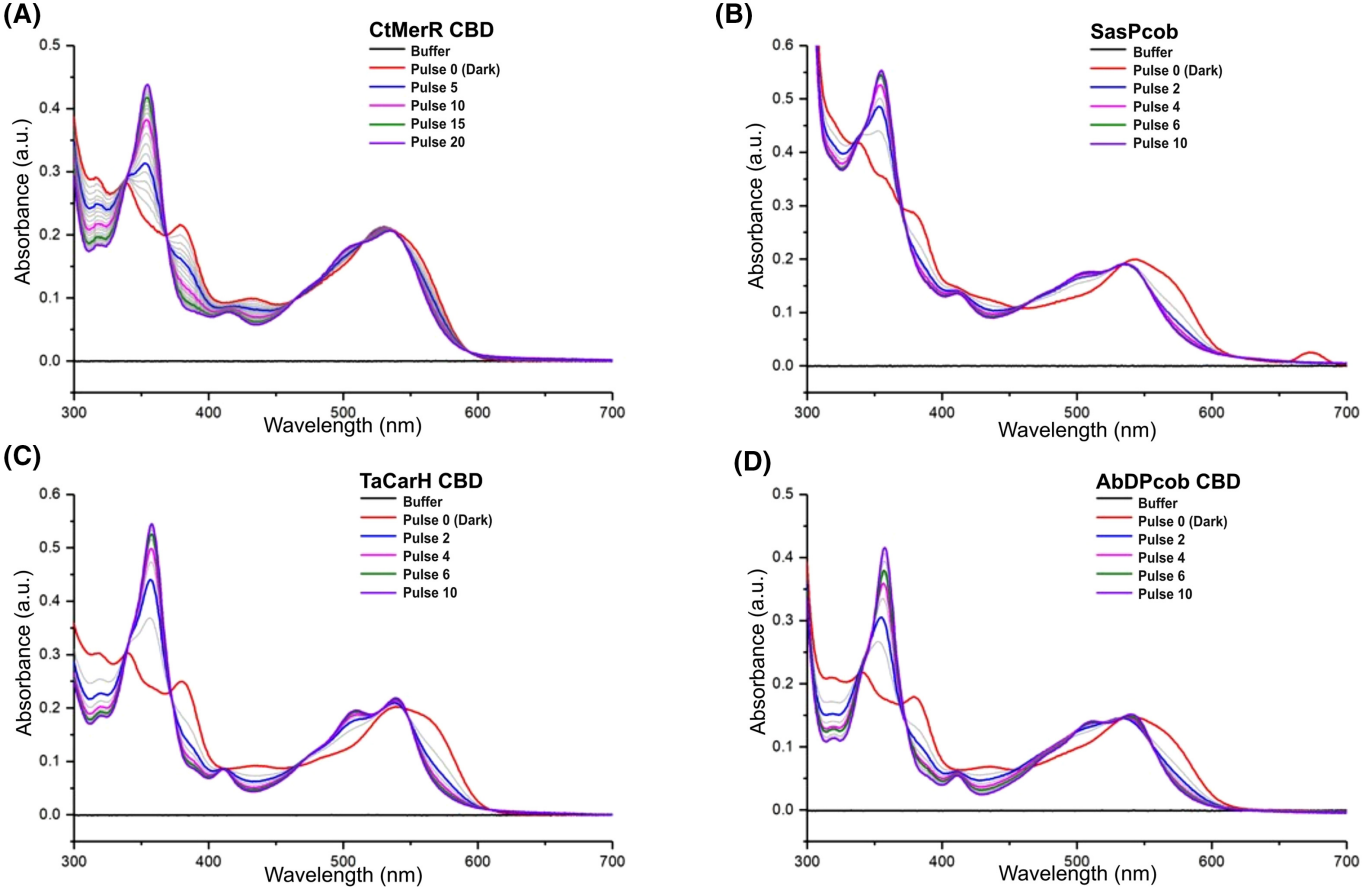
Spectral changes of *Ta*CarH_CBD, *Ct*MerR_CBD, *Sas*Pcob and *Ab*DPcob_CBD observed in response to green light. Absorbance spectra of *Ta*CarH_CBD (A), *Ct*MerR_CBD (B), *Sas*Pcob (C) and *Ab*DPcob_CBD (D) after a series of 120 ms 530 nm LED light pulses (*n* = 1). Each curve is coloured corresponding to the number of pulses of light (additional curves are shown in grey). Pulse 0 corresponds to the protein signal before light illumination. Graphs were plotted using origin 9.0 software (OriginLab, Northampton, MA, USA).

Encouraged by the *in silico* analysis, the selected proteins were expressed (Fig. S2) and their light response validated by absorbance spectroscopy measurements (Fig. 5; Fig. S3). For improved yield and spectral experiments, all relevant proteins were produced as truncated constructs with the DNA‐binding domain or the DGC domain removed (Fig. S2). *Ta*CarH, *Ct*MerR, *Sas*Pcob and *Ab*DPcob, all show a clear shift in the absorbance maximum compared to free AdoCbl indicating that AdoCbl is able to bind to these proteins. This is highlighted by the shift to 540 nm with a shoulder at 560 nm in the dark form (Fig. 5). In each case, upon illumination with green light, there is a decrease in absorbance at 560 nm and the appearance of a new peak at 356 nm. This indicates the formation of hydroxocobalamin (OHCbl) or a water‐ligated cobalamin, which forms after Co‐C bond photolysis and confirms that all these proteins respond to green light. It appears that *Ta*CarH, *Sas*Pcob and *Ab*DPcob require lower levels of illumination than *Ct*MerR to reach the final light state, suggesting that they have different quantum efficiencies that are likely to arise from different arrangements of the AdoCbl binding site. For protein *Ha*AI‐2E, which does not have the light response signature, only OHCbl binding was observed and neither adenosylcobalamin nor methylcobalamin (MeCbl) were able to bind (Fig. S3), indicating it is a light‐independent CBD. These spectroscopy measurements are consistent with the predictions from *SignatureFinder*. *Ha*AI‐2E may not be able to bind AdoCbl or MeCbl due to steric constraints or unfavourable interactions with nearby residues.

To investigate the oligomerisation state of the new photoreceptors in solution, we conducted analytical size exclusion chromatography (Fig. S4), multi‐angle light scattering (SEC‐MALS) (Fig. S5) and native mass spectrometry (Fig. S6) on the CBD of *Ab*DPcob, *Ct*MerR, *Ta*CarH and the full‐length *Sas*Pcob proteins. Analytical size exclusion indicated after light exposure the *Ab*DPcob CBD and *Sas*Pcob proteins did not change oligomerisation state whereas *Ct*MerR CBD and *Ta*CarH CBD did change oligomerisation state (Fig. S4). SEC‐MALS suggested *Ct*MerR and *Ta*CarH are stable as a tetramer in the dark and disassemble into monomers after light illumination, in a similar manner to *Tt*CarH. Surprisingly, *Sas*Pcob remains as a monomer in both states, whereas *Ab*DPcob forms a dimer in both dark and light conditions (Fig. S5). Though powerful analysis tools, SEC‐MALS and analytical size exclusion assume proteins are compact and spherical, and therefore, masses can be inaccurate [46, 47]. However, these results were consistent with those attained by native mass spectrometry for all proteins except *Ta*CarH. Interestingly, SEC‐MALS and analytical size exclusion calculated the protein mass to be approximately 13 kDa lower than the mass calculated from native mass spectrometry and the predicted mass from the FASTA sequence. This suggests this protein has a more compact conformation than a protein of this size typically has (Fig. S6).

Native mass spectrometry showed *Ta*CarH and *Ct*MerR form tetramers which monomerise in response to light. In addition, we observed the masses of the tetramers corresponded to the dark state with adenosylcobalamin bound and the light state with hydroxocobalamin bound (Fig. S6). This suggested that *Ct*MerR and *Ta*CarH are likely to control gene expression in a similar way to *Tt*CarH in response to light. However, *Sas*Pcob and *Ab*DPcob behave differently, and their function and behaviour regarding the light regulation required further investigation as discussed in our additional manuscript [29]. Interestingly, it was discovered that *Sas*Pcob does change oligomerisation state from a dimer to monomer in response to light when biliverdin is bound [29]. For all experiments described in this paper, biliverdin was not bound to any protein. Without biliverdin bound, we observed *Sas*Pcob was unable to dimerise and remained a monomer in the dark and light with slight changes in mass corresponding to adenosylcobalamin and hydroxocobalamin bound in the dark and light states, respectively (Fig. S6). The changes in oligomerisation state and the lack of stability observed by *Sas*Pcob and *Ab*DPcob without biliverdin bound suggests this cofactor is needed for proper protein folding, particularly in the dark state. All masses observed are summarised in Table S2.

### Structural analysis of conserved interactions responsible for light sensing in CBD

**Fig. 6:**
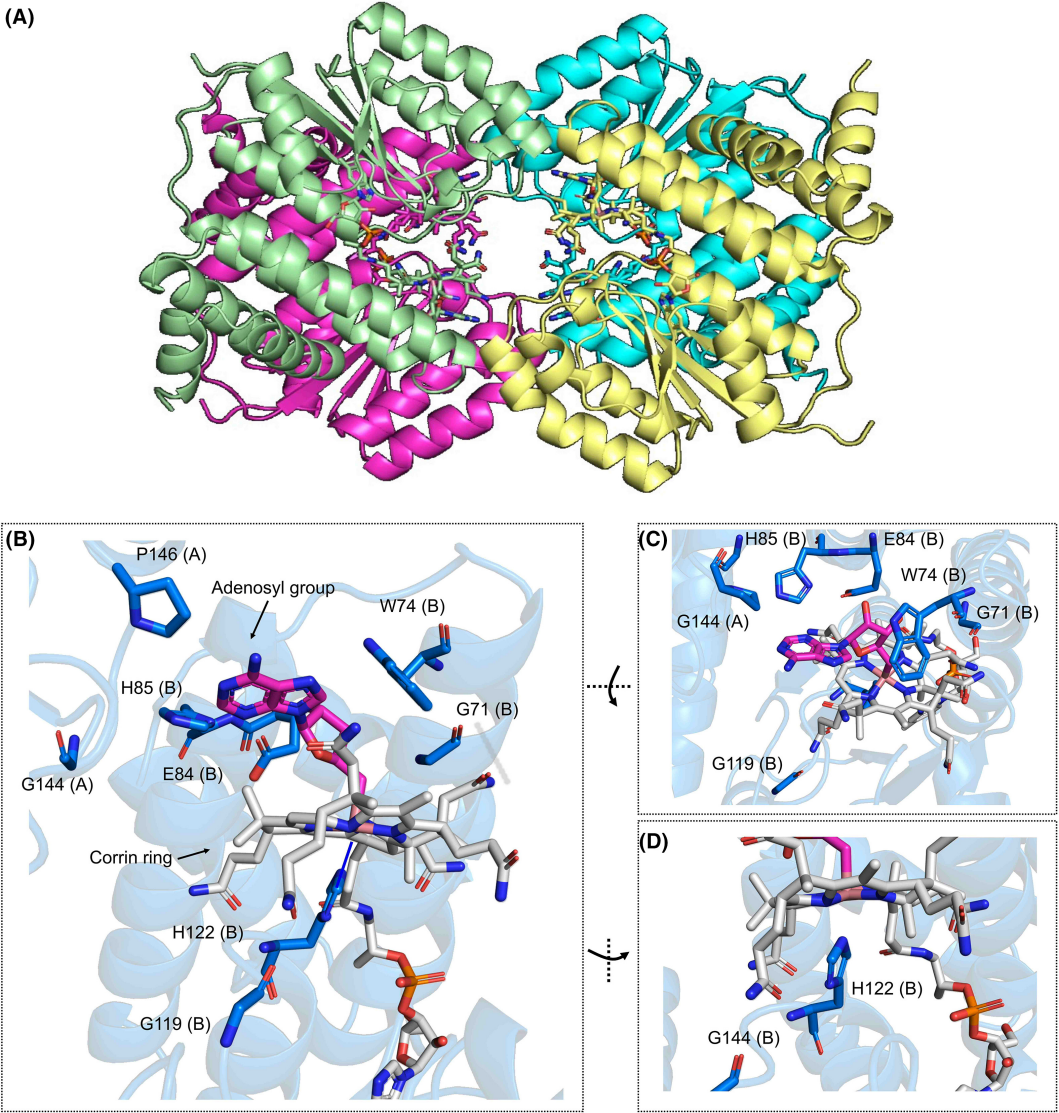
Interactions between light‐responsive signature sequences and adenosylcobalamin. Panel (A) shows the *Ct*MerR tetramer with each monomer in a different colour. AdoCbl molecules are shown as sticks in the colour representing the bound monomer. Panels (B–D) show the amino acids near the AdoCbl from the front view (B) top view (C) and side view (D) for the *Ct*MerR dark structure (PDB: 8JBS). The monomer each amino acid belongs to is designated by (A) or (B). Cobalamin is shown with sticks and balls in grey. The adenosyl group is coloured in magenta. All panels were made using PyMOL (Schrodinger Inc).

**Fig. 7:**
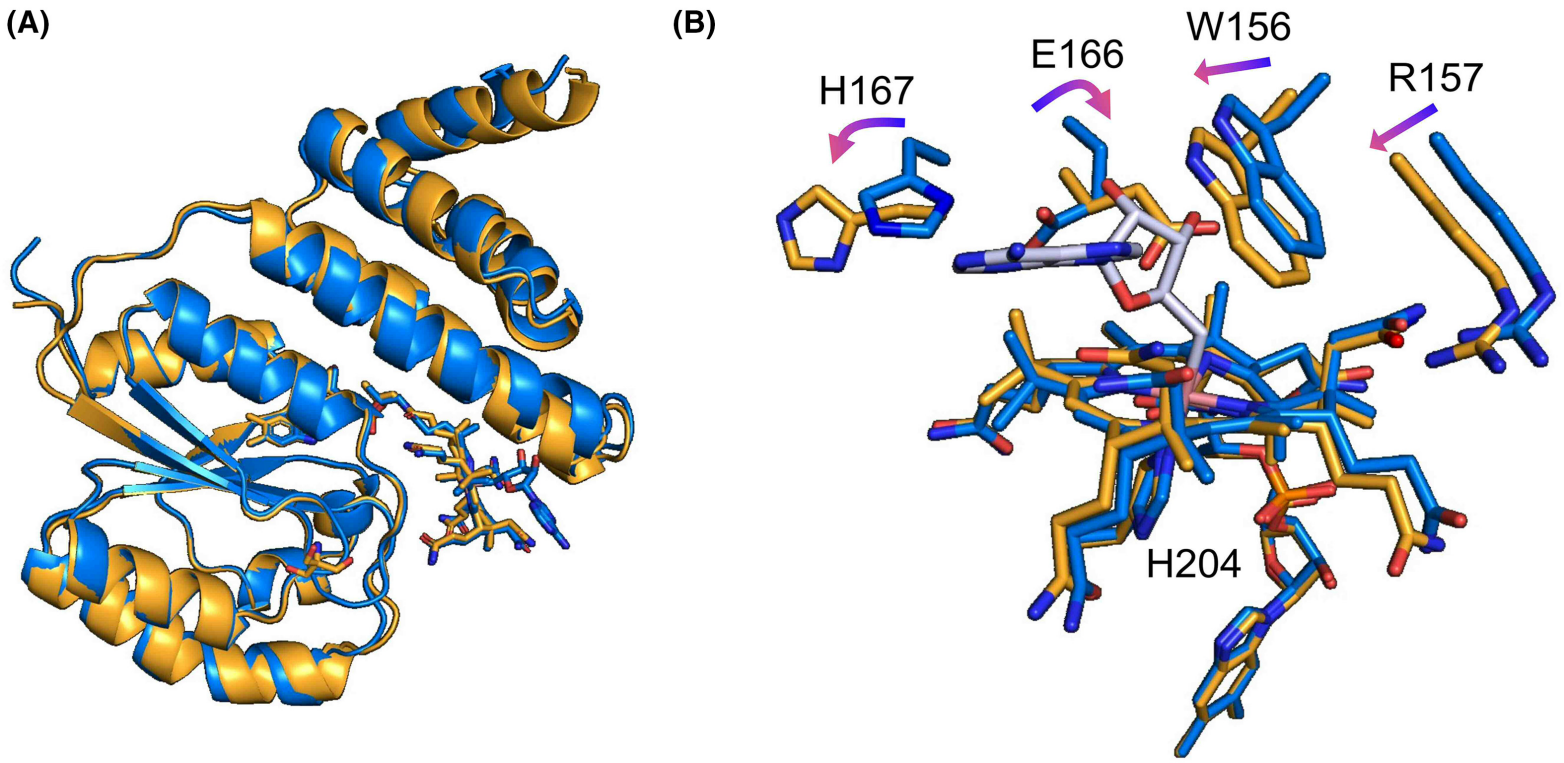
Crystal structures of *Ct*MerR and its comparison with *Tt*CarH. (A) Crystal structures of *Ct*MerR in the dark state (blue, 8JBS) and following anaerobic illumination (orange, 8JBT). (B) The movement of residues in response to light is shown for key residues with the dark state shown in blue and the anaerobic light state shown in orange. The adenosyl moiety is shown in grey. All panels were made using PyMOL (Schrodinger Inc).

**Fig. 8:**
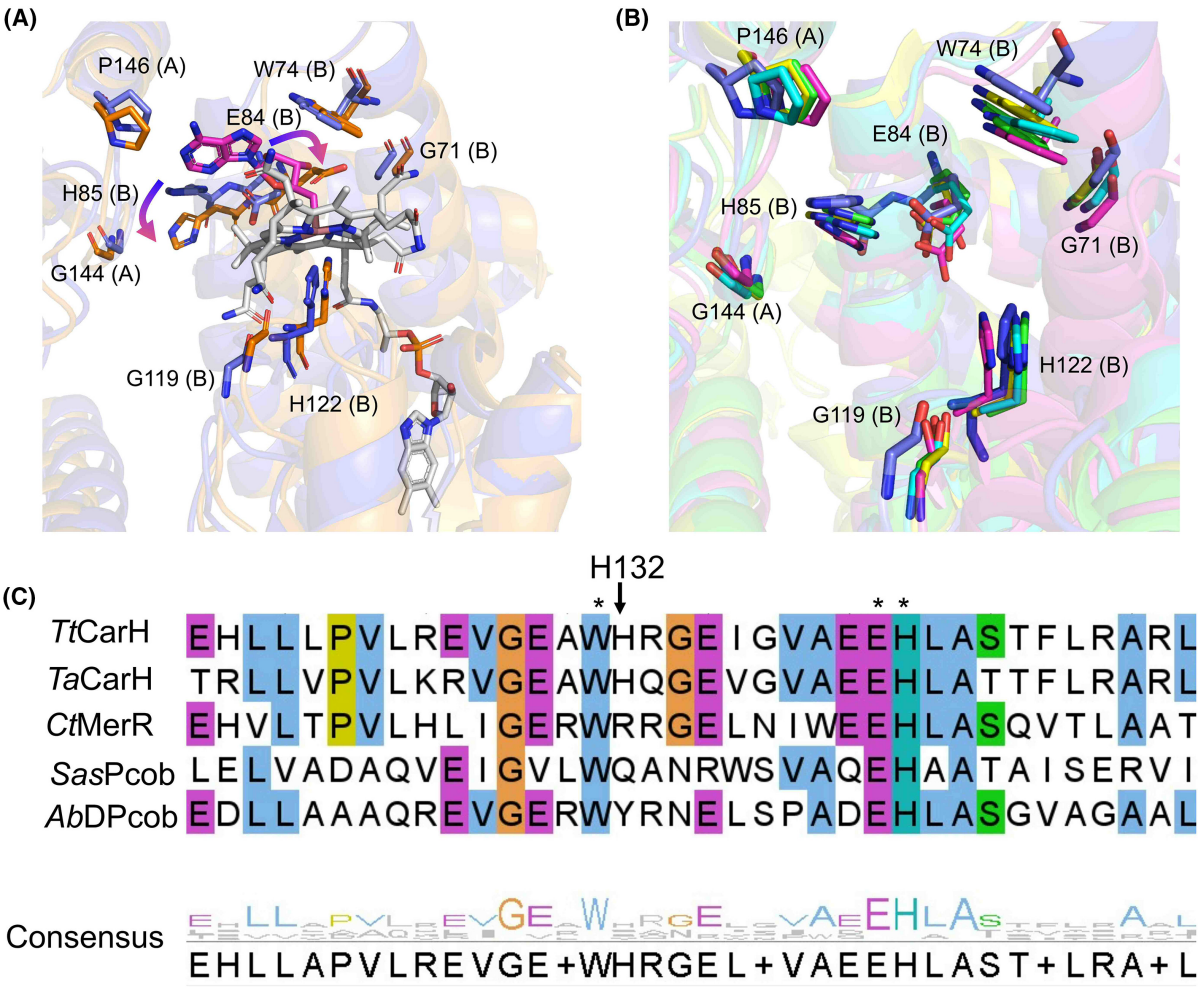
Structural characterisation of the interactions responsible for light sensing in the cobalamin‐binding pocket. (A) Rearrangements of signature residues upon illumination indicated by the alignment of crystal structures of *Ct*MerR in dark (blue) and light (orange) states show substantial movement. The monomer each amino acid belongs to is designated by (A) or (B). E84 flips so that the hydroxyl group interacts with the cobalt in the light state. The rearrangements of E84 and H85 are highlighted by arrows. Cbl is shown in sticks and balls in grey. (B) Alignment of signature residues of Alphafold models of *Sas*Pcob (cyan), *Ab*DPcob (magenta), *Ta*CarH (green) to crystal structures of *Tt*CarH (yellow) and *Ct*MerR (blue). The monomer each amino acid belongs to is shown in brackets next to their number. (C) Sequence alignment of residues in the cobalamin‐binding pocket of *Tt*CarH, *Ta*CarH, *Ct*MerR, *Sas*Pcob and *Ab*DPcob. Shared amino acid identities are labelled according to colour. Sequences are numbered according to the sequence of *Tt*CarH. The signature sequences are labelled with an asterisk. Sequence alignments were made using the MUSCLE tool from EMBL‐EBI. Structural panels were made using PyMOL (Schrodinger Inc). Accession codes are provided in Table S1.

Although it was not possible to obtain crystal structures for all the selected proteins, we were able to crystallise in the dark CBD domain of *Ct*MerR (Figs 6, 7; Table S3). *Ct*MerR crystallised in the P 3_1_ 2 1 space group with a dimer in the asymmetric unit. The functional tetramer can be generated with the symmetry elements (Fig. 6A). The structure also highlights how the identified light‐responsive signature elements (GxxW, EH, GxxH, GxxxP) interact with AdoCbl (Figs 6, 8; Fig. S7). His204 forms the lower axial ligand to the cobalt. Trp156 and Pro228 from the neighbouring monomer interact by hydrophobic interactions (Fig. 6A) while Glu84 forms a hydrogen bond (Fig. 6B) with the hydroxyl moiety of the upper adenosyl group. Main chain carbonyl groups Gly153 and Gly201 form hydrogen bonds with the corrin ring amide moieties (Fig. 6B,C). There are no obvious interactions between Gly226 and AdoCbl, although it presumably plays a role in modulation of the flexibility of the loop at the dimer interface. An alignment between the *Ct*MerR and *Tt*CarH crystal structures show the corrin ring and adenosyl group is accommodated by a conserved region formed by a helical bundle in the N‐terminal domain and the nearby Rossmann fold (Fig. S8), despite clear differences in more distant regions of the Rossmann fold structure.

Attempts to expose dark‐state crystals to light led to loss of diffraction, while light‐exposed protein would not crystallise. Light exposure of dark‐state crystals was undertaken in anaerobic conditions, following observations that WT and a variant forms of *Tt*CarH required oxygen for effective monomerisation resulting in large conformation changes that likely affect crystal packing [20]. For *Ct*MerR, light exposure under anaerobic conditions led to structural changes without monomerisation. The alpha helices within the Rossman fold show some movement when the protein is exposed to light, up to 2.0 Å around Q190 (Fig. 7A). However, the largest changes are present in the environment surrounding the AdoCbl cofactor. The dark and light‐adapted monomers overlaid with a r.m.s.d of 0.525 Å (calculated for all backbone Cɑ atoms). The cobalt‐carbon bond is cleaved, and the adenosyl moiety is absent from the structure leaving aquo/hydroxocobalamin bound (Fig. 7B). This is observed in *Tt*CarH [20] and *Sas*Pcob [29] upon light exposure suggesting a similar mechanism of photoactivation. Several key residues also rotate in this binding pocket including the signature residues E166 and H167.

The *Tt*CarH structural studies showed a horizontal shift of the cobalamin to form a bis‐His ligated cobalamin upon illumination [20]. However, this is not observed in the anaerobic *Ct*MerR light‐exposed structure, which contains an arginine rather than a histidine in the equivalent position (Fig. 8C). A similar observation is made for the *Tt*CarH H132A mutant, which undergoes modest structural rearrangement after light exposure but is unable to monomerise [48]. Sequence alignments show variation at this position; the equivalent residue for *Sas*Pcob is a glutamine and for *Ab*DPcob is a tyrosine (Fig. 8C). Instead of a bis‐His ligation to stabilise the cobalamin in the light state, a reorientation of the Glu166 residue in *Ct*MerR which allows formation of water‐mediated hydrogen bonding interaction with the cobalt (Fig. 7B). This suggests that the bis‐His ligated light state observed in *Tt*CarH is representative of the CBD light‐responsive state and that different photochemical mechanisms may be used in *Ct*MerR, *Sas*Pcob and *Ab*DPcob. Furthermore, the alignment of AlphaFold models of *Sas*Pcob, *Ab*DPcob and *Ta*CarH to the crystal structures of *Tt*CarH and *Ct*MerR highlighted a similar pattern of interactions with the adenosyl group of AdoCbl and the key signature residues (Fig. S8). The adenosyl group is vital for the nature of these proteins due to the cobalt–carbon bond cleavage induced by light resulting in structural rearrangement [48]. This provided further confirmation of the proposed signature for light sensing in CBD‐containing proteins.

### Database mining for further green‐light‐sensitive proteins

**Fig. 9:**
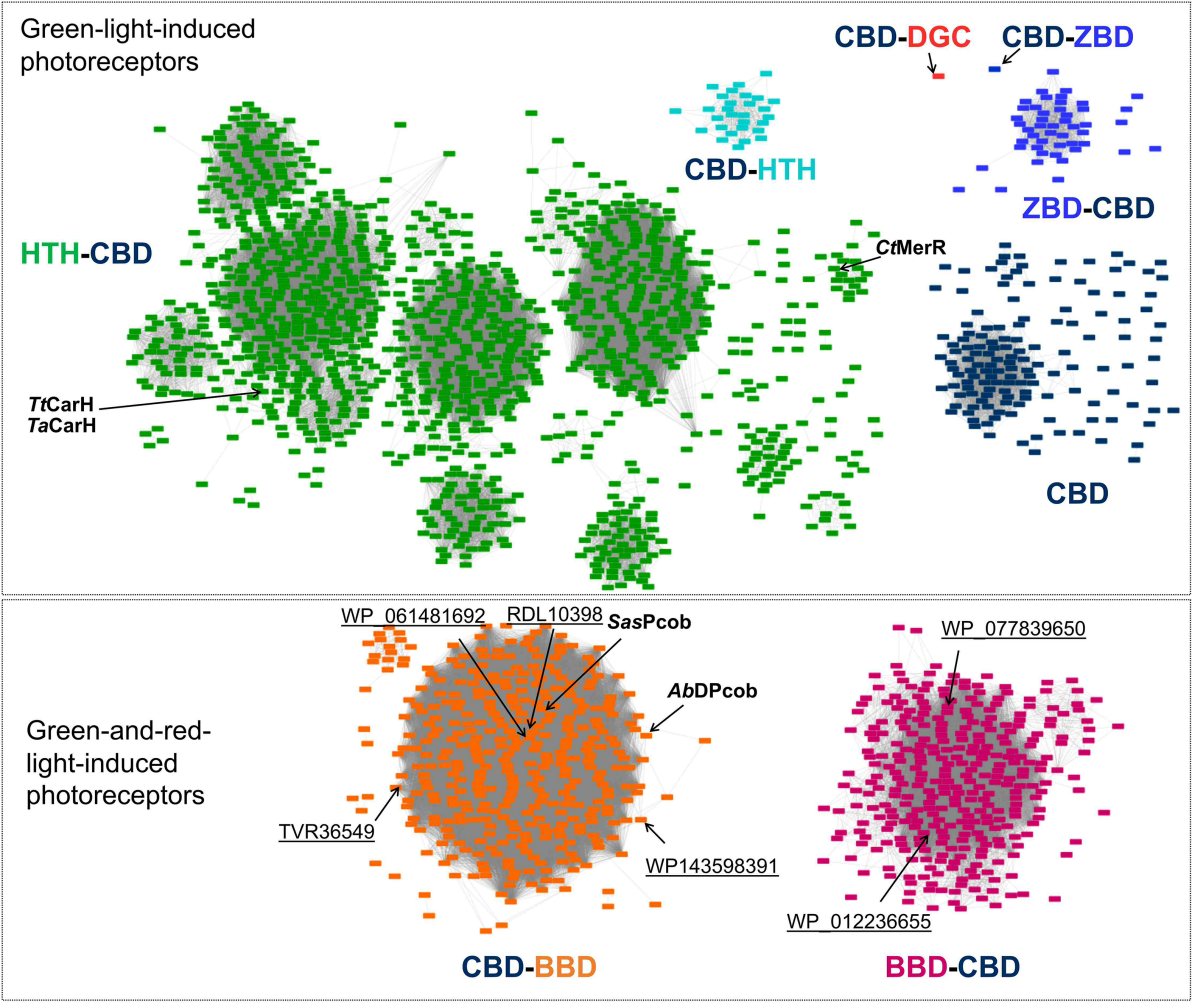
Sequence similarity network for putative green‐light‐responsive photoreceptors. Proteins containing cobalamin‐binding domains are labelled with underscored sequence entries to the InterPro database. ANTAR, AmiR and NasR transcription antitermination regulators; BBD, Biliverdin‐binding domain; CBD, Cobalamin‐binding domain; DBD, DNA‐binding domain; DGC, Diguanylate cyclase; DICT, diguanylate cyclases and phosphodiesterases and two‐component systems; GAF, cGMP‐specific phosphodiesterases, adenylyl cyclases and FhlA; HTH, Helix‐Turn‐Helix; MEDS, MEthanogen/methylotroph DcmR Sensory; PDE, Phosphodiesterase. For Alphafold models of each cluster, see Fig. S9.

The light‐responsive CBD signature was used to search for further green‐light‐sensitive proteins in the public database. In this analysis, 1561 out of 128 000 CBD‐containing sequences (InterPro database entry: IPR006158) were found to have the proposed signature after clustering using a sequence identity of 80%. The sequence similarity network was constructed to show the diversity of the sequences (Fig. 9). A 3D model from each cluster was predicted by AlphaFold, and the putative functional domains were annotated according to the model (Fig. S9). The new photoreceptors *Ct*MerR, *Sas*Pcob and *Ab*DPcob identified in the current work and the previously reported CBD‐containing photoglobins [49] were also retrieved from the public database by using this approach, further highlighting the effectiveness of the signature as a filter for retrieving light‐responsive CBDs. Light‐responsive CBDs can be found fused to output domains such as helix‐turn‐helix (HTH) domains, diguanylate cyclase (DGC) domain, biliverdin‐binding domain (BBD), GAF domains and MEthanogen/methylotroph DcmR Sensory (MEDS) at both the N‐terminal and C‐terminal region, indicating it is a versatile building block for multi‐domain proteins to regulate manifold activities inside cells in response to light. Interestingly, database mining revealed more BBD‐CBD fusion proteins, the recently discovered photoglobin family, thus expanding the toolbox of dual‐light‐regulated photoreceptors [49]. The interactive sequence similarity network for identifying novel green‐light‐induced photoreceptors is now accessible to the public.

## Conclusions

As the only characterised green‐light‐induced B_12_‐dependent photoreceptor, CarH has shown exciting potential in light‐dependent biotechnological applications [24, 25, 26, 27]. The discovery of new green‐light‐sensitive photoreceptors to diversify and expand the optogenetics toolbox is now a major bottleneck in this process. Although (Ado)Cbl is the chromophore used by CarH for sensing green light, not all cobalamin‐binding proteins are photoreceptors and the characteristic features responsible for photoreception are currently unknown. We have now developed a computational workflow, *SignatureFinder*, to identify the signature for binding a ligand of interest by combining structural and sequence analysis of input sequences, which facilitates further database mining of the resulting signature. The power of this approach has been demonstrated by applying *SignatureFinder* to identify light‐responsive signatures (GxxW, EH, GxxH, GxxxP) in CBD‐containing proteins, which was then validated by spectroscopic and structural analysis. Additionally, we characterised a new family of proteins, called the photocobilins, containing adenosylcobalamin and biliverdin [29]. Encouraged by these results, *SignatureFinder* has now been constructed into a web server freely accessible to the public and is expected to find signature sequences for binding any ligand of interest, such as adenosylcobalamin. Using *SignatureFinder*, we have identified many more possible green‐light‐sensitive proteins that could be used for future optogenetics applications and have published the sequence similarity network on NEDx, which has a user‐friendly interface that is freely accessible. The tools and information provided in this study will save a huge amount of time and cost in the discovery of new photoreceptors to significantly expand the optogenetics toolbox.

## Materials and methods

### Signaturefinder workflow

The *Signaturefinder* workflow is based on the combination of homology modelling, molecular docking, sequence alignment and phylogenetic tree construction. The details for each step are described below:

#### Homology modelling

A three‐dimensional structure for each sequence was built by using MODELLER [50]. The structure from the protein data bank with the highest sequence identity was used as a template for constructing the model. Every model was aligned by PyMol to the input reference structure and root‐mean‐square deviation (*RMSD*) values between each model and the reference structure were computed using the Cα atoms. An *RMSD* threshold was set for picking sequences that have similar structures to the reference structure. Outliers were removed from further data processing. Outliers were identified as proteins with RMSD values over 2.0 Å between the models and reference.

#### Molecular docking

The ligand from the input reference structure was extracted. The centre of mass of the ligand was used as the centre of the docking box. The docking box size was calculated by using script *eBoxSize.pl* [51] considering the gyration radius of the substrate, which improves the docking accuracy [51]. In the case of CBDs, a cubic docking box with the putative models were aligned to the reference structure and the ligand was docked to the docking box. Molecular docking was carried out by AutoDock vina [52] with exhaustiveness value 10.

#### Sequence alignment

MUSCLE [53] was used to produce sequence alignments for the putative sequences. The conservation of each site was viewed by Jalview [44].

#### Phylogenetic tree

MUSCLE [53] was used to make sequence alignments for the input sequences. Raxml‐ng [54] based on maximum likelihood (ML) methods was used to calculate the phylogenetic tree for the input sequence. Popular protein evolutionary models DAYHOFF, DCMUT, LG, JTT, MTREV, WAG, RTREV, CPREV, VT, BLOSUM62 and MTMAM were evaluated and the best model which gives the lowest AIC (Akaike Information Criterion) score were used to generate the final tree for the input sequences [55]. The best tree out of 100 ML trees in newick tree format were used to view the evolutionary relationship of all sequences by ITOL [42].

### SignatureFinder web server

The web server *SignatureFinder* is accessible to the public by https://enzymeevolver.com/SignatureFinder. The example input files are attached as supplementary files.

### Protein expression and purification

All chemicals were ordered from Sigma‐Aldrich (St. Louis, MI, USA). The selected genes were synthesised by GeneArt (Thermo Fisher, Loughborough, UK) and subcloned into the pET21a vector (MilliporeSigma (Novagen), Malvern, UK). The recombinant plasmids were then transformed into *E. coli* BL21(DE3) cells for protein expression. Transformed cells were grown in auto induction LB medium (Formedium™, glucose/lactose ratio 1:4) containing 50 μg·mL^−1^ ampicillin for 24 h at 25 °C. Cells were harvested by centrifugation at 6000 ***g*** for 10 min at 4 °C, resuspended in lysis buffer (20 mm HEPES pH 7.0, 500 mm NaCl, 25 mm imidazole) supplemented with protease inhibitor cocktail and lysed by a cell disruptor (Constant Systems). The cell lysate was centrifuged at 51 000 ***g*** for 1 h at 4 °C to remove cell debris. The soluble cell lysate was loaded onto a His‐trap column, and bound protein was eluted with elution buffer (20 mm HEPES pH 7.0, 500 mm NaCl, 250 mm imidazole). The peak fractions were collected and incubated with AdoCbl for at least 2 h. The sample was then loaded onto the size exclusion column (HiLoad 16/600 Superdex 200) for further purification and removal of free ligands. The protein fractions with ligand bound were collected for further experiment.

### Light titration with 530 nm LED

The absorbance spectra of all the samples were collected using a Cary 50 spectrophotometer (Agilent Technologies, Cheadle, UK). The TDS3032C 300 MHz Digital Phosphor Oscilloscope (Tektronix, Bracknell, UK) and TGP110 10 MHz Pulse Generator with Delay (Thurlby Thandar Instruments, Huntingdon, UK) were used to generate a 120 ms 530 nm LED (Thorlabs Inc. Ely, UK) pulse. After each LED pulse, the spectrum was collected until there were no further significant changes in absorbance. The experiment was carried out under a dim red light to prevent any light illumination of the sample.

### Sec‐MALS

Size exclusion chromatography coupled with multi‐angle light scattering was carried out to investigate the oligomeric state of new photoreceptors. For analysis of light‐activated oligomeric changes, protein was analysed in the dark. Light‐exposed samples were exposed for 5 min using a 530 nm LED as described above. MiniDAWN TREOS MALS detector and Optilab rEX refractive index meter (Wyatt, Santa Barbara, CA, USA) were used to collect light scattering signals. An Agilent G7110B HPLC pump, degasser and autoinjector (Agilent, Santa Clara, CA, USA) was used to auto load the samples (50 μL each run, 1 mg·mL^−1^). For size exclusion separation, a Superdex 200 10/300 GL column using 20 mm HEPES, pH 6.8, 150 mm NaCl buffer at 1 mL·min^−1^. All results were processed according to referenced protocol [47]. Raw data were exported and plotted using Origin 9.0 software (OriginLab, Northampton, MA, USA). Further details are available in [29].

### Analytical size exclusion

Protein was purified as detailed previously and kept at −80°C before analysis. Samples were defrosted, diluted to 20 μm and 500 μL loaded onto a Superdex 200 pg 10/300 GL column from Cytiva Life Sciences. Protein was exposed to ambient light for 10 min at room temperature. The column was equilibrated in 20 mm HEPES, 150 mm NaCl pH 8. The mass of the protein was determined from a calibration curve generated using two molecular weight kits. The first was a Gel Filtration Calibration Kit from Cytiva Life Sciences containing Ferritin (440 000 Da), Aldolase (158 000 Da), Conalbumin (75 000 Da), Ovalbumin (44 000 Da), Carbonic Anhydrase (29 000 Da) and Ribonuclease A (13 700 Da). The second was the HPLC Protein Molecular Weight Markers kit from Sigma‐Aldrich containing cytochrome c (12,400 Da), myokinase (32 000 Da), enolase (67 000 Da) lactate dehydrogenase (142 000 Da) and glutamate dehydrogenase (290 000 Da).

### Native mass spectrometry

Protein was analysed as previously described [48]. Briefly, protein was desalted into 200 mm ammonium acetate pH 8.0. NanoESI capillaries were prepared in house from thin‐walled borosilicate capillaries (inner diameter 0.9 mm, outer diameter 1.2 mm) (World Precision Instruments, Hitchin, UK) using a Flaming/Brown P‐97 micropipette puller (Sutter Instrument Company, Hitchin, UK). Native MS data were acquired using the Thermo Scientific Q Exactive Hybrid Quadrupole‐Orbitrap mass spectrometer (Thermo Fisher Scientific, Loughborough, UK). For all spectra generated, the spray current was kept between 0.2 and 0.3 μA with the spray voltage varying between 0.9 and 1.3 kV accordingly. The resolution used was 25 000 for all proteins except *Ab*DPcob which used 12 500 due to high salt and instability of the protein. For MS settings, see previous work [48]. Proteins were exposed to ambient light for 5 min for light‐exposed data collection. For analysis, 5 min spectra were averaged and processed in Thermo Xcalibur (Thermo Fisher Scientific, Loughborough, UK) before further data analysis with UniDec [56].

### Protein crystallisation, data collection and structure determination

The protein after size exclusion was concentrated to 10 mg·mL^−1^ in 20 mm HEPES pH 7.5, 150 mm NaCl. Crystallisation was performed using the sitting drop vapour diffusion technique (200 nL crystallisation reagent mixed with 200 nL protein). The dark state of *Ct*MerR CBD crystals was obtained from 0.1 m Amino acids, 0.1 m Bicine and Tris pH 8.5, 50% v/v Glycerol and Poly(ethylene glycol) 4000. To obtain the light state structure of *Ct*MerR CBD, the dark crystals were transferred into glove box and degassed for at least 3 days. Both crystals were cryo protected with the addition of 20% PEG 200 to the reservoir solution prior to flash cooling in liquid nitrogen. Individual datasets were collected from single cryo‐protected crystals at beamlines i03, i04 and i04‐1 (Diamond Light Source). All data were indexed, scaled and subsequently integrated with Xia2 [57]. Structure determination was initially performed by molecular replacement in Phaser [58] using a search model generated by AlphaFold2 [45]. A combination of automated and manual rebuilding and refinement in Refmac [59] and COOT [60] were used to produce the refined models. Validation with both Molprobity [61] and PDB_REDO [62] was integrated into the iterative rebuild process. Complete data collection and refinement statistics are available in Table S3. The atomic coordinates and experimental data have been deposited in the Protein Data Bank (www.pdb.org). All figures were made using open‐source PyMOL software (Version 3.0, Schrödinger, LLC, New York, USA).

## Conflict of interest

The authors declare no conflict of interest.

## Author contributions

NSS, DL and DJH initiated project. NSS and SZ coordinated the project. YY, AH and LJ constructed *SignatureFinder* workflow and the web server. YY, LJ, and FD performed bioinformatics analysis and produced the online SignatureFinder tool. LNJ, SZ, YY and MS performed protein purification. SZ and YY performed spectroscopy experiment, SEC and crystallisation experiment. LNJ performed native spectrometry and analytical gel filtration experiments. SZ and HP performed and analysed the structural elucidation of CtMerR. LNJ and YY wrote the manuscript and generated the figures. All authors discussed the results and commented on the manuscript.

### Peer review

The peer review history for this article is available at https://www.webofscience.com/api/gateway/wos/peer‐review/10.1111/febs.17377.

## Supporting information


**Fig. S1.** Comparison of the Alphafold modelled HaAI‐2E structure with *Tt*CarH.
**Fig. S2**. SDS‐PAGE gels for WP_053768024, WP_157850694, WP_033429474, HAS09818 and WP_052573826.
**Fig. S3**. Response of WP_052573826 (*Ha*AI‐2E) to green light by absorbance spectroscopy experiment.
**Fig. S4**. Analytical size exclusion chromatography for *Ta*CarH, *Ct*MerR, *Sas*Pcob and *Ab*DPcob.
**Fig. S5**. SEC‐MALS for *Ta*CarH, *Ct*MerR, *Sas*Pcob and *Ab*DPcob.
**Fig. S6**. Native MS for *Tt*CarH, *Ta*CarH, *Ct*MerR, *Sas*Pcob and *Ab*DPcob.
**Fig. S7**. Electron density map of *Ct*MerR crystals.
**Fig. S8**. Structural comparison of *Tt*CarH and *Ct*MerR.
**Fig. S9**. Full length AlphaFold models for representative clusters in sequence similarity networks of putative light‐responsive CBD‐containing proteins.
**Table S1**. Comparison of sequences identified using *SignatureFinder*.
**Table S2**. Summary of masses observed for novel B12‐binding proteins.
**Table S3**. Data collection and refinement statistics for CtCBD.

## Data Availability

The data supporting these results are available within the article and the Supporting Information.
