# Supplementary material for: SignatureFinder enables sequence mining to identify cobalamin‐dependent photoreceptor proteins
Source: FEBS J. 2024 Dec 24;292(3):635–52. doi: 10.1111/febs.17377 (PMC11796333; doi:10.1111/febs.17377)
Supplement: Supplementary file 1 — Fig. S1. Comparison of the Alphafold modelled HaAI‐2E structure with TtCarH. Fig. S2. SDS‐PAGE gels for WP_053768024, WP_157850694, WP_033429474, HAS09818 and WP_052573826. Fig. S3. Response of WP_052573826 (HaAI‐2E) to green light by absorbance spectroscopy experiment. Fig. S4. Analytical size exclusion chromatography for TaCarH, CtMerR, SasPcob and AbDPcob. Fig. S5. SEC‐MALS for TaCarH, CtMerR, SasPcob and AbDPcob. Fig. S6. Native MS for TtCarH, TaCarH, CtMerR, SasPcob and AbDPcob. Fig. S7. Electron density map of CtMerR crystals. Fig. S8. Structural comparison of TtCarH and CtMerR. Fig. S9. Full length AlphaFold models for representative clusters in sequence similarity networks of putative light‐responsive CBD‐containing proteins. Table S1. Comparison of sequences identified using SignatureFinder. Table S2. Summary of masses observed for novel B12‐binding proteins. Table S3. Data collection and refinement statistics for CtCBD. [file FEBS-292-635-s001.pdf]

SignatureFinder enables sequence mining to identify cobalamin-dependent photoreceptor proteins

Yuqi Yu[1,2‡], Laura N. Jeffreys[1‡], Harshwardhan Poddar[1], Adam Hill[3], Linus Johannissen[1], Fanzhuo Dai[1], Michiyo Sakuma[1], David Leys[1], Derren J. Heyes[1], Shaowei Zhang[1,4, *], Nigel S. Scrutton[1, *]

[1]Department of Chemistry, The University of Manchester, Manchester Institute of Biotechnology, 131 Princess Street, Manchester, M1 7DN, UK.

[2]Current address: Astra Zeneca, Francis Crick Avenue, Cambridge CB2 0QH, UK

[3]Department of Chemistry, The University of Manchester, Dover Street Building, Oxford Road, Manchester M13 9PL, UK

[4]Current address: Department of Biology and Chemistry, College of Sciences, National University of Defense Technology, Changsha, China.

‡ These authors contributed equally to this work

[*]Corresponding authors: Professor Nigel Scrutton, Nigel.Scrutton@manchester.ac.uk; Dr Shaowei Zhang, shaowei.zhang@nudt.edu.cn

Running title: The signature of cobalamin photoreceptor proteins

Abbreviations: Cbl, cobalamin; CBD, Cobalamin-Binding Domain; HTH, Helix-Turn-Helix; DBD, DNA-binding Domain; BBD, Biliverdin-Binding Domain; DGC, Diguanylate Cyclase; MEDS, MEthanogen/methylotroph DcmR Sensory; PDE, Phosphodiesterase; GAF, cGMP-specific phosphodiesterases, Adenylyl cyclases and FhlA; ANTAR, AmiR and NasR transcription antitermination regulators; DICT, DIguanylate Cyclases and phosphodiesterases and Two-component systems; SSN, sequence similarity network; RMSD, root mean square deviation

Keywords: photoreceptors, cobalamin, bioinformatics, structure, sequence motif

Conflicts of Interest: The authors declare no conflict of interest.

Supplementary Tables

Supplementary Table 1 – Comparison of sequences identified using *SignatureFinder*. CBDs that structurally resemble *Tt*CarH, light-independent enzymes and the light-sensitive flavoprotein AppA are coloured in dark green, and orange, respectively. Proteins predicted to be light insensitive are shown in blue such as methionine synthase. Each sequence is labelled by using the code from NCBI database.

Sequence	Database annotation	Under same group with *Tt*CarH in phylogenetic tree?	Template protein used	Structural similarity RMSD to *Tt*CarH (Å)	*SignatureFinder* annotation
WP_083499352	cobalamin-dependent protein	Yes	TtCarH	0.9	CBD-containing photoreceptor
WP_022798111	MerR family transcriptional regulator	Yes	TtCarH	0.8	CBD-containing photoreceptor
WP_053768024	MerR family transcriptional regulator	Yes	TtCarH	0.3	CBD-containing photoreceptor
WP_110886267	MerR family transcriptional regulator	Yes	TtCarH	1.0	CBD-containing photoreceptor
MBK9714687	B12-binding domain-containing protein	Yes	TtCarH	0.5	CBD-containing photoreceptor
MBS1964747	B12-binding domain-containing protein	Yes	TtCarH	0.5	CBD-containing photoreceptor
WP_029595673	MerR family transcriptional regulator	Yes	TtCarH	0.7	CBD-containing photoreceptor
WP_040375569	MerR family transcriptional regulator	Yes	TtCarH	0.6	CBD-containing photoreceptor
MBC7645486	MerR family transcriptional regulator	Yes	TtCarH	0.9	CBD-containing photoreceptor
WP_207499832	MerR family transcriptional regulator	Yes	TtCarH	0.8	CBD-containing photoreceptor
MBS1148846	CarH	Yes	TtCarH	0.7	CBD-containing photoreceptor
MBL8910852	cobalamin B12-binding domain-containing protein	Yes	TtCarH	1.0	CBD-containing photoreceptor
WP_141956459	MerR family transcriptional regulator	Yes	TtCarH	0.6	CBD-containing photoreceptor
WP_158288580	MerR family transcriptional regulator	Yes	TtCarH	0.7	CBD-containing photoreceptor
WP_018411532	MerR family transcriptional regulator	Yes	TtCarH	0.3	CBD-containing photoreceptor
WP_108283697	cobalamin-dependent protein	Yes	TtCarH	0.7	CBD-containing photoreceptor
WP_119358590	MerR family transcriptional regulator	Yes	TtCarH	0.6	CBD-containing photoreceptor

WP_030588653	cobalamin B12-binding domain-containing protein	No	TtCarH	1.8	CBD-containing photoreceptor
WP_157850694	B12-binding domain-containing protein	No	TtCarH	0.7	CBD-containing photoreceptor
WP_033429474	cobalamin-dependent protein	No	TtCarH	1.3	CBD-containing photoreceptor
HAS09818	hypothetical protein DCS55_04760	No	TtCarH	1.0	CBD-containing photoreceptor
WP_143598391	diguanylate cyclase	No	TtCarH	0.6	CBD-containing photoreceptor
WP_018529170	cobalamin-dependent protein	No	TtCarH	0.6	CBD-containing photoreceptor
NNM96001	cobalamin-binding protein	No	TtCarH	1.3	CBD-containing photoreceptor
MBW4030475	hypothetical protein HIU57_07330	No	TtCarH	0.7	CBD-containing photoreceptor
MBW4077952	hypothetical protein HIU84_05470	No	TtCarH	0.6	CBD-containing photoreceptor
TVR19749	hypothetical protein EA387_12895	No	TtCarH	0.9	CBD-containing photoreceptor
TVP71310	hypothetical protein EA340_04835	No	TtCarH	0.5	CBD-containing photoreceptor
MBK8469870	cobalamin-dependent protein	No	TtCarH	0.6	CBD-containing photoreceptor
WP_158648004	cobalamin-dependent protein	No	TtCarH	0.7	CBD-containing photoreceptor
WP_162794426	cobalamin-dependent protein	No	TtCarH	0.7	CBD-containing photoreceptor
WP_162799307	cobalamin-dependent protein	No	TtCarH	0.7	CBD-containing photoreceptor
A8XY95	Methionine synthase	No	1bmtA	3.2	methionine synthase
Q9I2Q2	Methionine synthase	No	1bmtA	3.1	methionine synthase
Q9KUW9	Methionine synthase	No	1bmtA	3.2	methionine synthase
WP_143807391	methionine synthase	No	1bmtA	3.9	methionine synthase
Q87L95	Methionine synthase	No	1bmtA	4	methionine synthase
WP_099044311	methionine synthase	No	1bmtA	3.3	methionine synthase
WP_125354568	methionine synthase	No	1bmtA	3.1	methionine synthase
WP_026464863	cobalamin-dependent protein	No	1bmtA	3.9	methionine synthase
P11653	Methylmalonyl-CoA mutase	No	1reqA	10.7	Methylmalonyl-CoA mutase
WP_008515145	cobalamin-dependent protein	No	1xrsB	7.7	D-lysine 5,6-aminomutase alpha subunit

WP_012056435	cobalamin-dependent protein	No	1xrsB	7.8	D-lysine 5,6-aminomutase alpha subunit
WP_132039050	cobalamin-dependent protein	No	1xrsB	8	D-lysine 5,6-aminomutase alpha subunit
E3PRJ5	Lysine aminomutase	No	1xrsB	8	D-lysine 5,6-aminomutase alpha subunit
WP_209661149	cobalamin-dependent protein	No	1xrsB	8	D-lysine 5,6-aminomutase alpha subunit
Q8TS71	Dimethylamine corrinoid protein 2	No	1y80A	2	Predicted cobalamin binding protein
WP_012194551	corrinoid protein	No	1y80A	2.8	Predicted cobalamin binding protein
WP_064974560	corrinoid protein	No	1y80A	3	Predicted cobalamin binding protein
WP_007784990	corrinoid protein	No	1y80A	2.8	Predicted cobalamin binding protein
WP_013275879	corrinoid protein	No	1y80A	3.6	Predicted cobalamin binding protein
WP_015049525	corrinoid protein	No	1y80A	2.9	Predicted cobalamin binding protein
G5EDY2	Tryptophanyl-tRNA synthetase	No	1y80A	5.1	Predicted cobalamin binding protein
WP_054937972	corrinoid protein	No	1y80A	3	Predicted cobalamin binding protein
P58983	Trimethylamine corrinoid protein 2	No	1y80A	1.3	Predicted cobalamin binding protein
P58981	Dimethylamine corrinoid protein 3	No	1y80A	1.3	Predicted cobalamin binding protein
WP_013899222	methanol--corrinoid protein MtaC	No	2i2xB	9	Methyltransferase 1
WP_042684257	methyltransferase cognate corrinoid protein	No	2i2xB	8.3	Methyltransferase 1
WP_096711372	methanol--corrinoid protein MtaC	No	2i2xB	8.9	Methyltransferase 1
WP_048166109	methanol--corrinoid protein MtaC	No	2i2xB	9	Methyltransferase 1
Q46EH3	Methanol--corrinoid protein co-methyltransferase	No	2i2xB	9.1	Methyltransferase 1
WP_048117314	methanol--corrinoid protein MtaC	No	2i2xB	9.1	Methyltransferase 1
WP_081728522	cobalamin-dependent protein	No	2i2xB	13.3	Methyltransferase 1

MBA2280799	cobalamin B12-binding domain-containing protein	No	2i2xB	9.5	Methyltransferase 1
Q23381	Probable methylmalonyl-CoA mutase	No	2xijA	12.1	methylmalonyl-CoA mutase, mitochondrial
P22033	Methylmalonyl-CoA mutase	No	2xijA	15.4	methylmalonyl-CoA mutase, mitochondrial
XP_015264376	methylmalonyl-CoA mutase	No	2xijA	12.4	methylmalonyl-CoA mutase, mitochondrial
Q9GK13	Methylmalonyl-CoA mutase	No	2xijA	5.9	methylmalonyl-CoA mutase, mitochondrial
P13009	Methionine synthase	No	3bulA	9.1	methionine synthase
WP_072801850	methylmalonyl-CoA mutase	No	6oxcA	7.3	Methylmalonyl-CoA mutase large subunit
P9WJK5	Probable methylmalonyl-CoA mutase	No	6oxcA	3.5	Methylmalonyl-CoA mutase large subunit
WP_047323553	methylmalonyl-CoA mutase	No	6oxcA	8.6	Methylmalonyl-CoA mutase large subunit
Q9P9L5	Monomethylamine corrinoid protein 2	No	3ezxA	4.2	Monomethylamine corrinoid protein 1
WP_036797394	BLUF domain-containing protein	No	4hh0A	3.4	AppA protein
WP_011909988	BLUF domain-containing protein	No	4hh0A	10.1	AppA protein
WP_190788861	BLUF domain-containing protein	No	4hh0A	9.4	AppA protein
WP_067602093	BLUF domain-containing protein	No	4hh0A	8.3	AppA protein
WP_090196164	BLUF domain-containing protein	No	4hh0A	13.1	AppA protein
WP_111993280	BLUF domain-containing protein	No	4hh0A	4.3	AppA protein

Supplementary Table S2 – Summary of masses observed for novel B12-binding proteins.

**Protein**	**Analytical size exclusion chromatography**		**Native MS**				**SEC-MALS**		**Predicted mass without cofactor (kDa)**
	**Approx Dark mass (kDa)**	**Approx Light Mass (kDa)**	**Dark Mass (kDa)**	**Predominant charge**	**Light Mass (kDa)**	**Predominant charge**	**Dark mass (kDa)**	**Light Mass (kDa)**	
***Ab*DPcob CBD**	75.0	75.0	75.6	+11	72.2	+11	35.1 ± 0.01	35.5 ± 0.04	36.2
***Sas*Pcob**	40.0	40.0	38.4	+11	38.1	+12	36.3 ± 0.01	37.5 ± 0.32	37.5
***Ct*MerR CBD**	110.0	25.0	115.9	+22	28.5	+10	109.8 ± 0.93	29.7 ± 0.97	27.3
***Ta*CarH CBD**	90.0	25.0	100.5	+19	24.3	+9	86.6 ± 0.18	21.9 ± 0.81	23.1

Supplementary Table S3 - Data collection and refinement statistics for CtCBD.

	*Ct*MerR CBD dark<br>PDB: 8JBS	*Ct*MerR CBD light<br>PDB: 8JBT
**Data collection**		
Space group	P 31 2 1	P 31 2 1
Cell dimensions		
a, *b*, *c* (Å)	125.218 125.218 73.0879	123.485 123.485 73.1045
α, β, γ (°)	90 90 120	90 90 120
Resolution (Å)	108.4 - 2.3<br>(2.382 - 2.3)*	106.9 - 2.299<br>(2.382 - 2.299)
R_{merge}	0.1589 (2.659)	0.2803 (2.463)
$I / \sigma I$	12.50 (0.62)	8.08 (0.72)
Completeness (%)	99.83 (98.91)	99.93 (99.89)
Redundancy	20.6 (19.5)	20.6 (19.4)
**Refinement**		
Resolution (Å)	108.4 - 2.3	106.9 - 2.299
No. reflections	29627 (2906)	27458 (2862)
R_{work} / R_{free}	0.1910/0.2224	0.2064/0.2253
No. atoms		
Protein	3588	3601
Ligand/ion	418	236
Water	41	66
B-factors		
Protein	63.95	39.41
Ligand/ion	49.19	39.76
Water	49.52	37.22
R.m.s. deviations		
Bond lengths (Å)	0.014	0.010
Bond angles (°)	2.08	1.74

*Values in parentheses are for highest-resolution shell.

Supplementary Figures

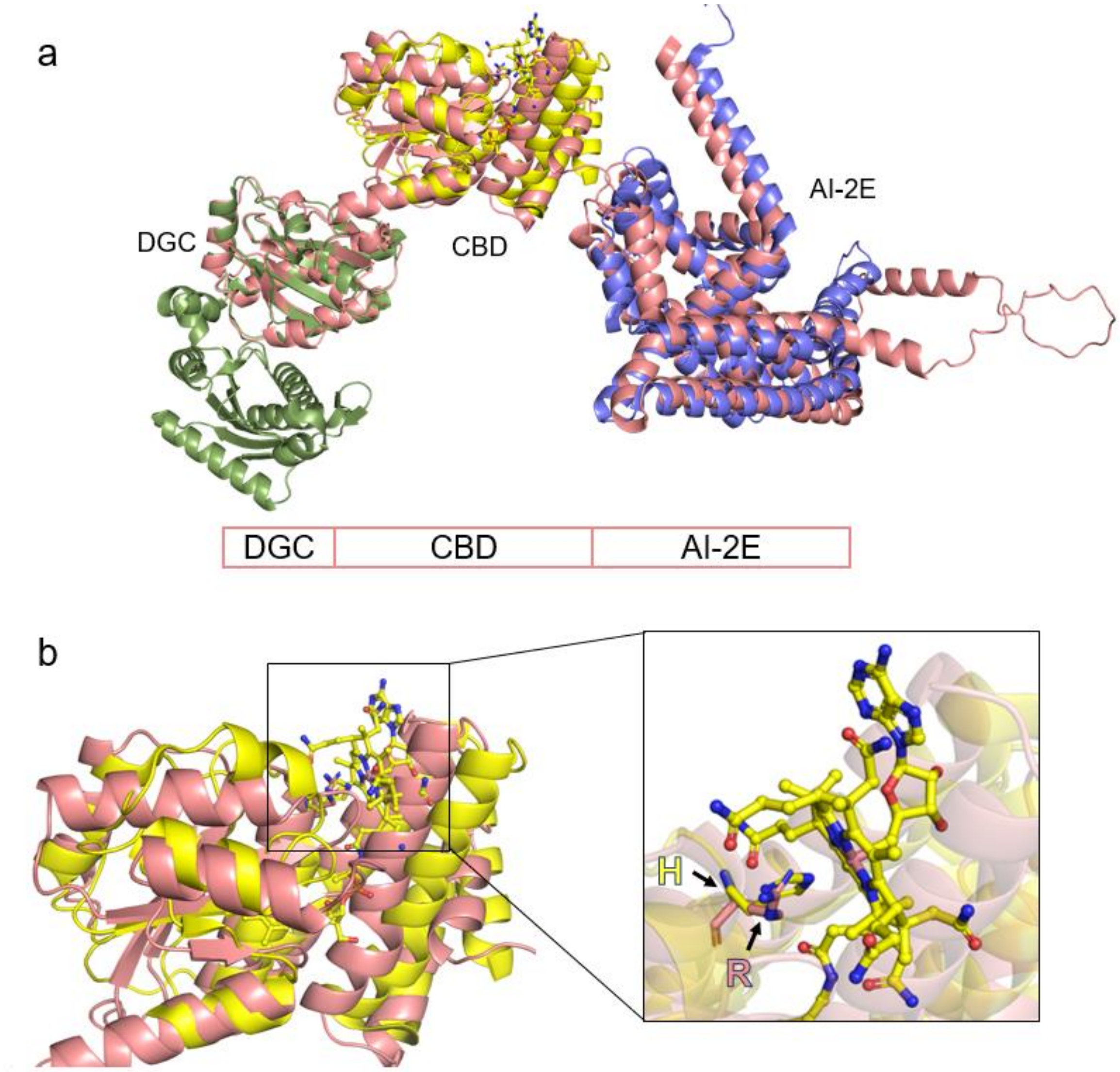

Supplementary Figure S1 - Comparison of the Alphafold modelled HaAI-2E structure with *Tt*CarH. (a) Alphafold modelled structure of *Ha*AI-2E (WP_052573826) in pink aligned to a quorum-sensing signal autoinducer-2 exporter (AI-2E) (PDB: 7NB6) in purple, *Tt*CarH (PDB: 5C8E) in yellow and a diguanylate cyclase (PDB: 4ZMU) in green. (b) CBD of *Ha*AI-2E aligned to *Tt*CarH (PDB: 5C8A) (in yellow). The Arg residue in *Ha*AI-2E and equivalent His177 residue in *Tt*CarH were shown in sticks. The AdoCbl is shown in sticks and balls. All panels were made using PyMOL (Schrodinger Inc).

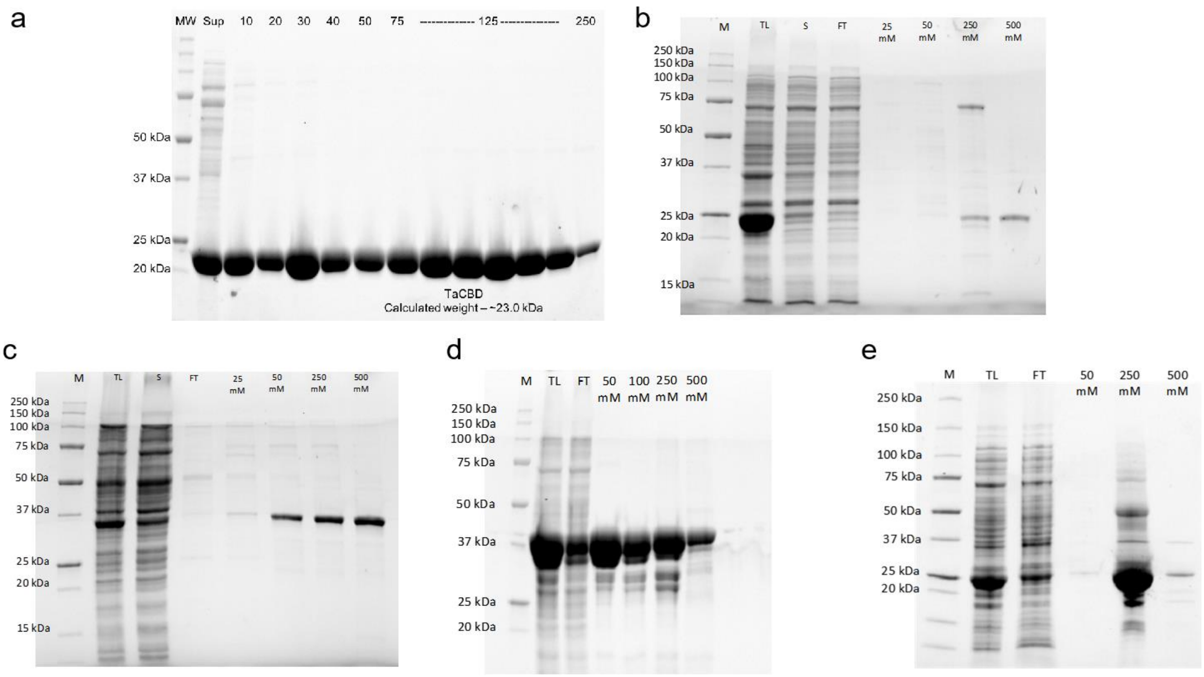

Supplementary Figure S2 - SDS-PAGE gels for WP_053768024, WP_157850694, WP_033429474, HAS09818 and WP_052573826. Protein expression in *E. coli* BL21 (DE3) with auto induction LB medium (FormediumTM, glucose/lactose ratio 1:4) containing 50 µg/mL ampicillin for 24 h at 25 °C. Target proteins were purified through His-trap affinity binding. Proteins eluted with different imidazole concentration were collected and examined by SDS-PAGE gels.

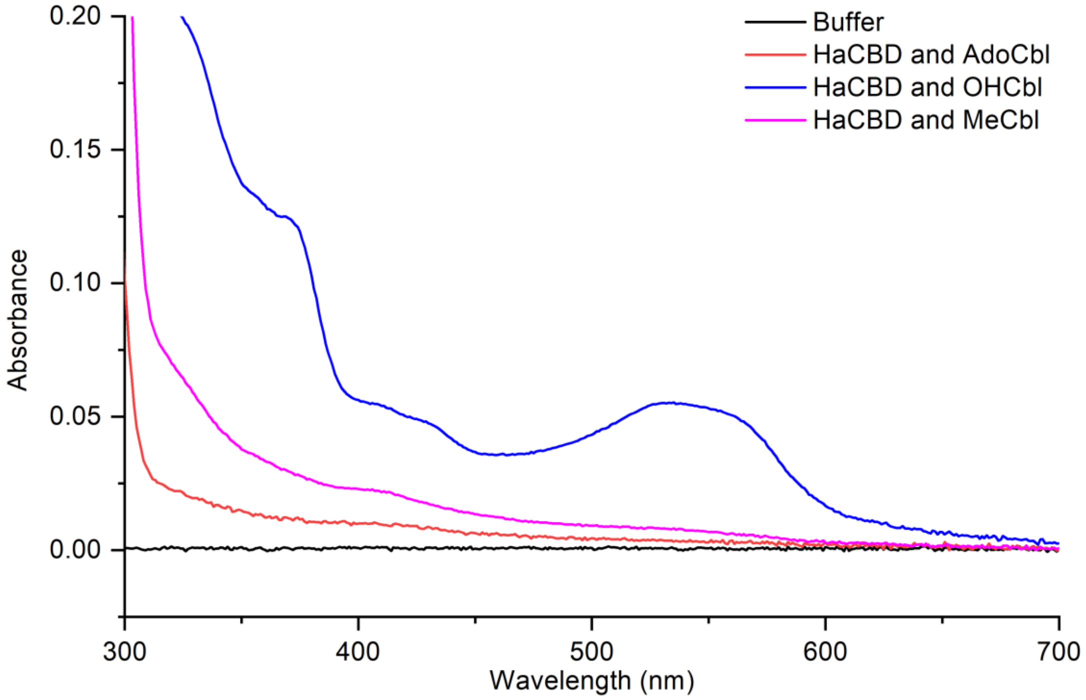

Supplementary Figure S3 - Response of WP_052573826 (*Ha*AI-2E) to green light by absorbance spectroscopy experiment. Cobalamin-Binding Domain of *Ha*AI-2E (*Ha*CBD) was purified and incubated with different cobalamins. After running through desalting column, only OHCbl shows binding with HaCBD. All samples were exposed to 530nm LED light and their absorbance spectra were recorded and shown in the figure. Graph was plotted using Origin 9.0 software (OriginLab, Northampton, MA).

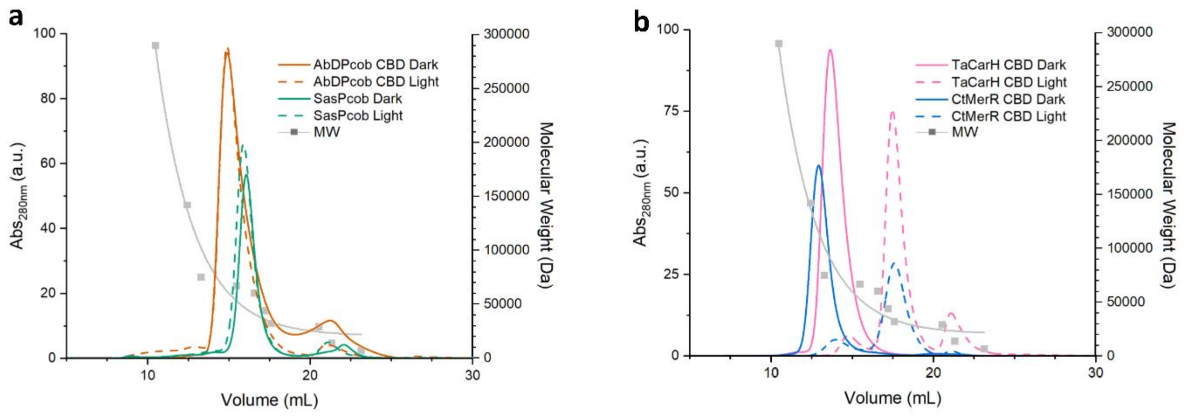

Supplementary Figure S4 – Analytical size exclusion chromatography for *Ta*CarH, *Ct*MerR, *Sas*Pcob and *Ab*DPcob. Proteins were analysed on a Superdex 200 10/300 GL column providing approximate masses for dark and light states. Graphs were plotted using Origin 9.0 software (OriginLab, Northampton, MA).

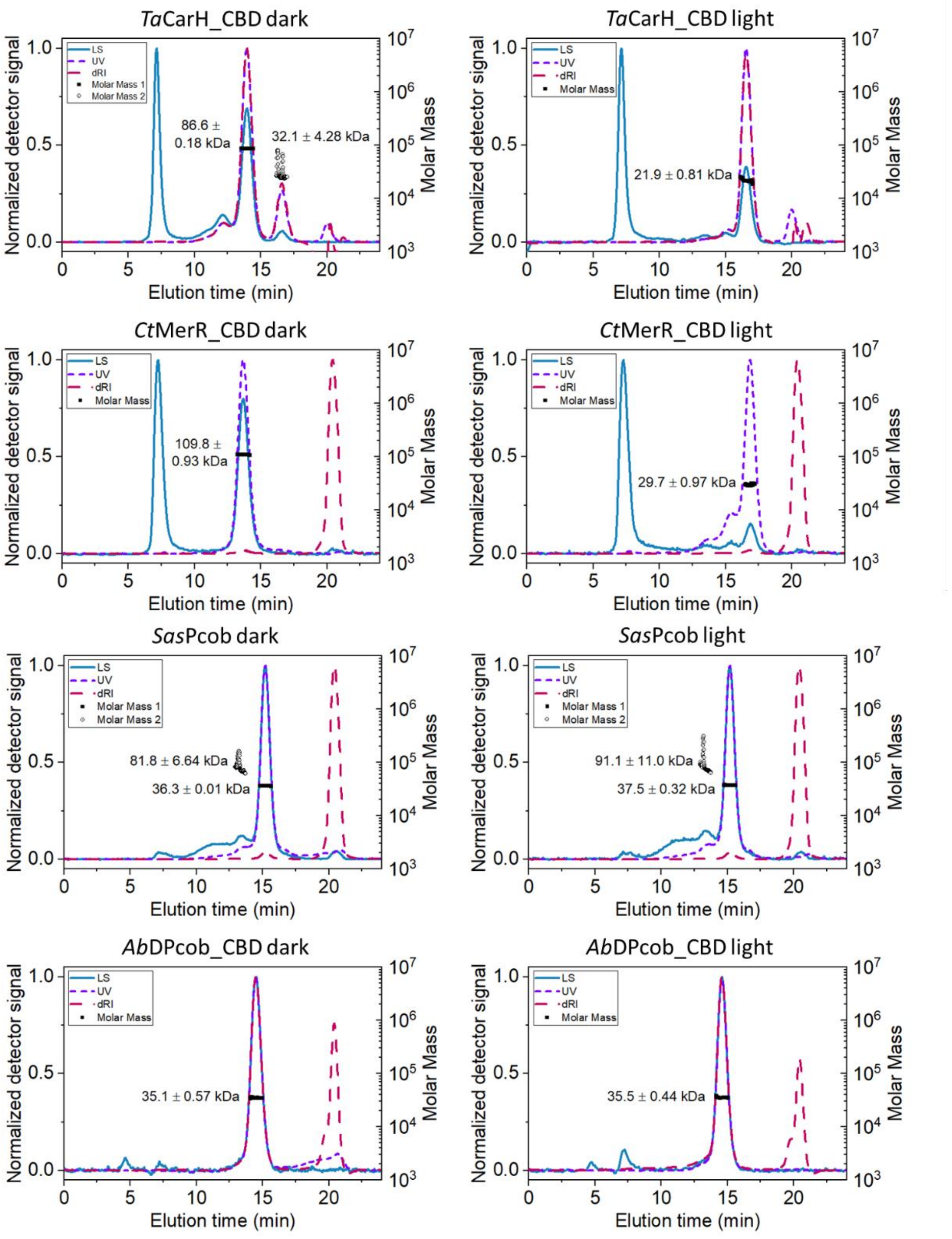

Supplementary Figure S5 - SEC-MALS for *Ta*CarH, *Ct*MerR, *Sas*Pcob and *Ab*DPcob. SEC-MALS chromatograms are shown for the samples under dark and light condition, Chromatogram traces of light scattering (LS), ultraviolet (UV) and differential refractive index (dRI) signals are shown in the figure as solid or dashed line. Molar mass for main peaks is plotted as square scatters. The estimated molecular weight of the main fraction is labelled in the figure. Graphs were plotted using Origin 9.0 software (OriginLab, Northampton, MA).

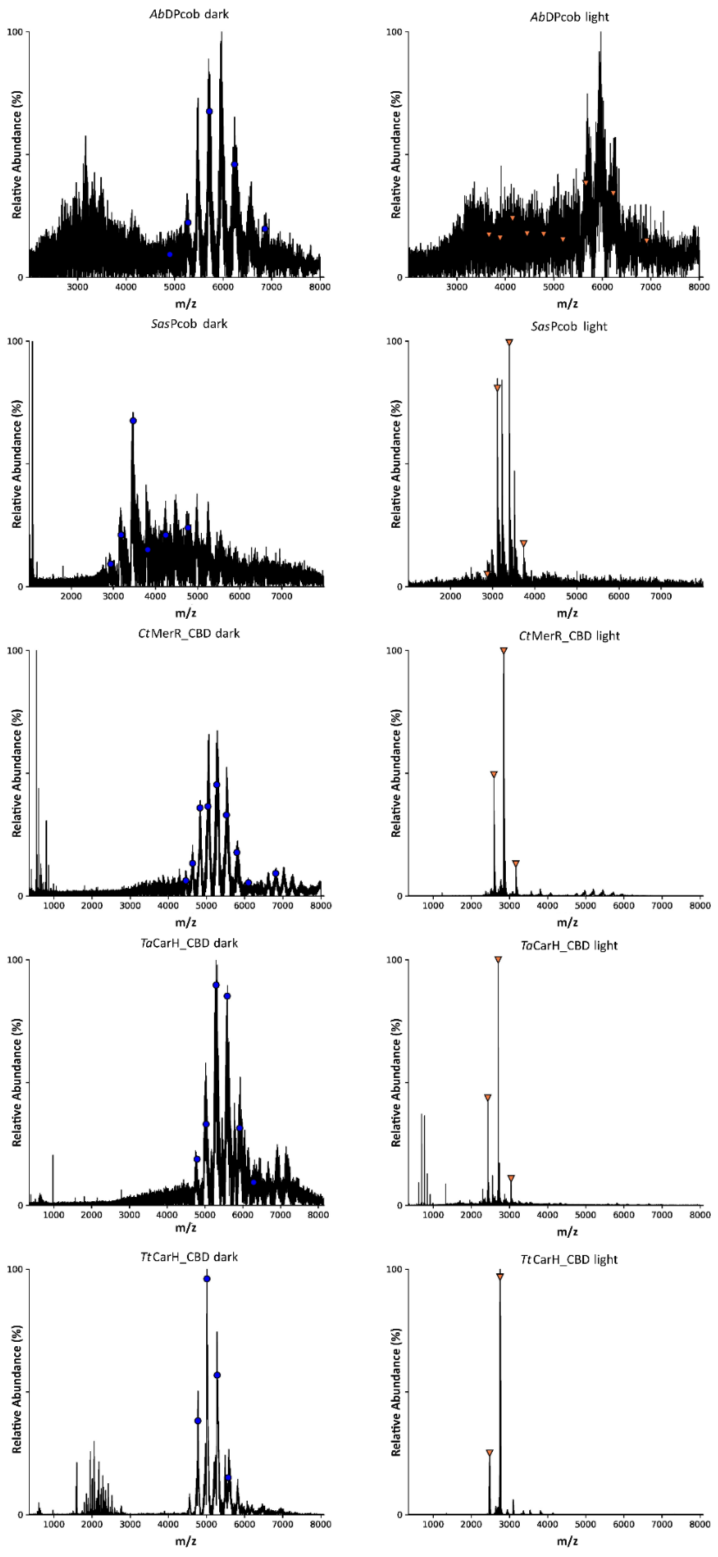

Supplementary Figure S6 – Native MS for *Tt*CarH, *Ta*CarH, *Ct*MerR, *Sas*Pcob and *Ab*DPcob. Spectra are shown for the samples under dark and light condition. The predominant peaks in each spectrum corresponding to the protein are labelled with blue circles for dark or orange triangles for light exposed samples. The masses are shown in Supplementary Table 2. *Ab*DPcob and *Sas*Pcob were not stable in ionisation buffers without the biliverdin cofactor. Graphs were produced using UniDec.

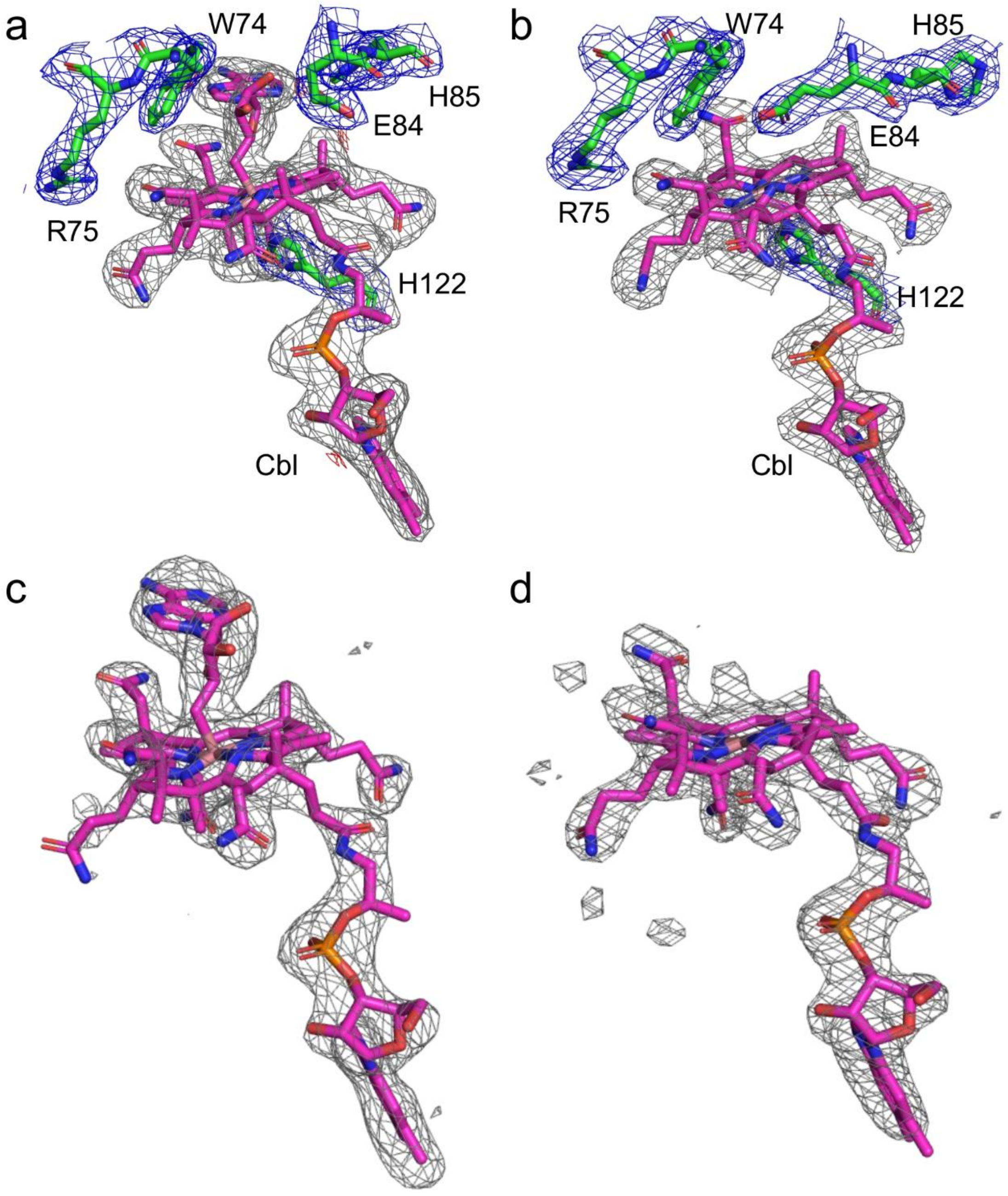

Supplementary Figure S7 – Electron density map of *Ct*MerR crystals. Electron density maps of key residues around Cbl molecule in dark (**a**) and anaerobic light (**b**) state crystals. Cbl is shown in magenta and surrounding residues around are shown in green. $2F_o{-}F_c$ maps are contoured at 1σ and coloured as grey and blue for the Cbl molecule and surrounding residues respectively. Cbl $F_o{-}Fc$ omit maps are contoured at 3σ (in grey) in dark (**c**) and anaerobic light (**d**) state crystals. All panels were made using PyMOL (Schrodinger Inc).

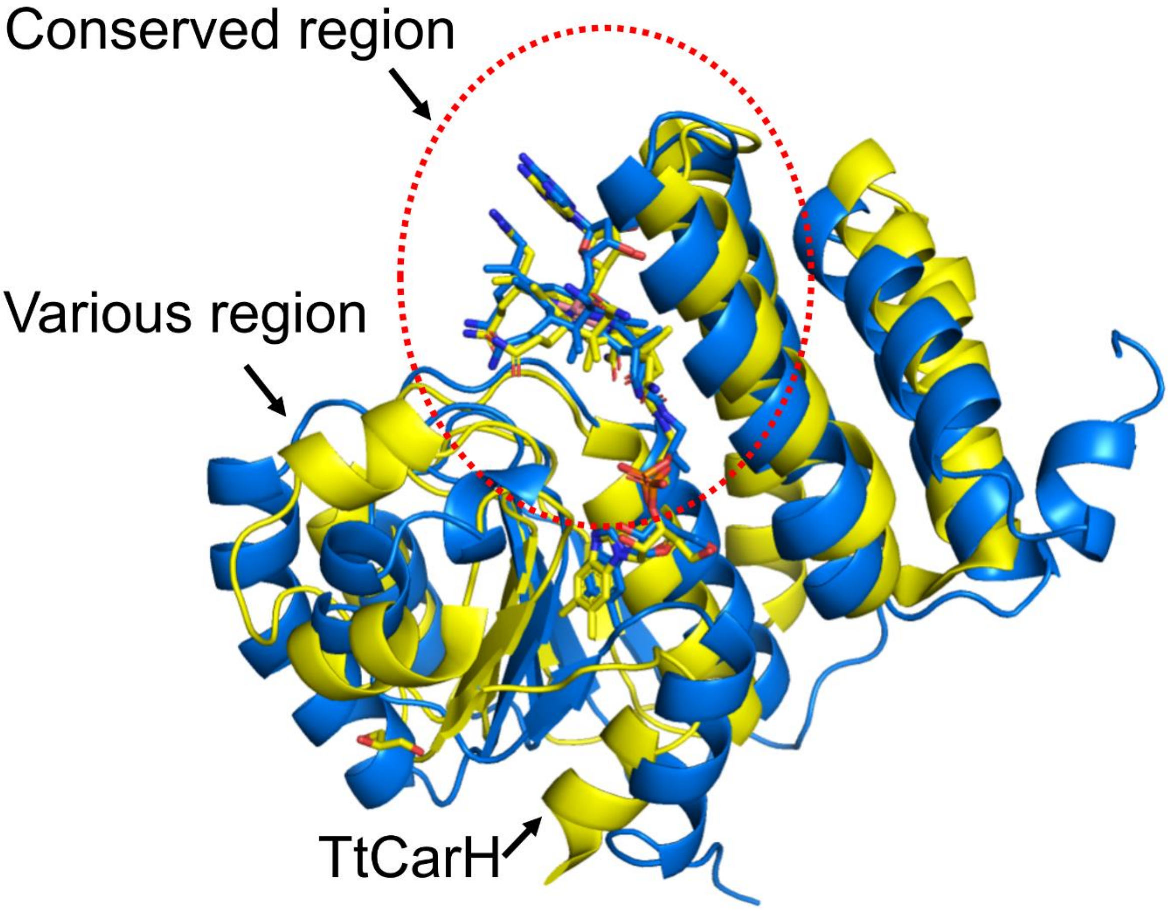

Supplementary Figure S8 – Structural comparison of *Tt*CarH and *Ct*MerR. The dark state of *Tt*CarH (PDB 5C8A) is shown in yellow and the dark state of *Ct*MerR (PDB 8JBS) is shown in blue. The conserved upper ligand binding region and various Rossmann fold region are highlighted in red. Figure was made using PyMOL (Schrodinger Inc).

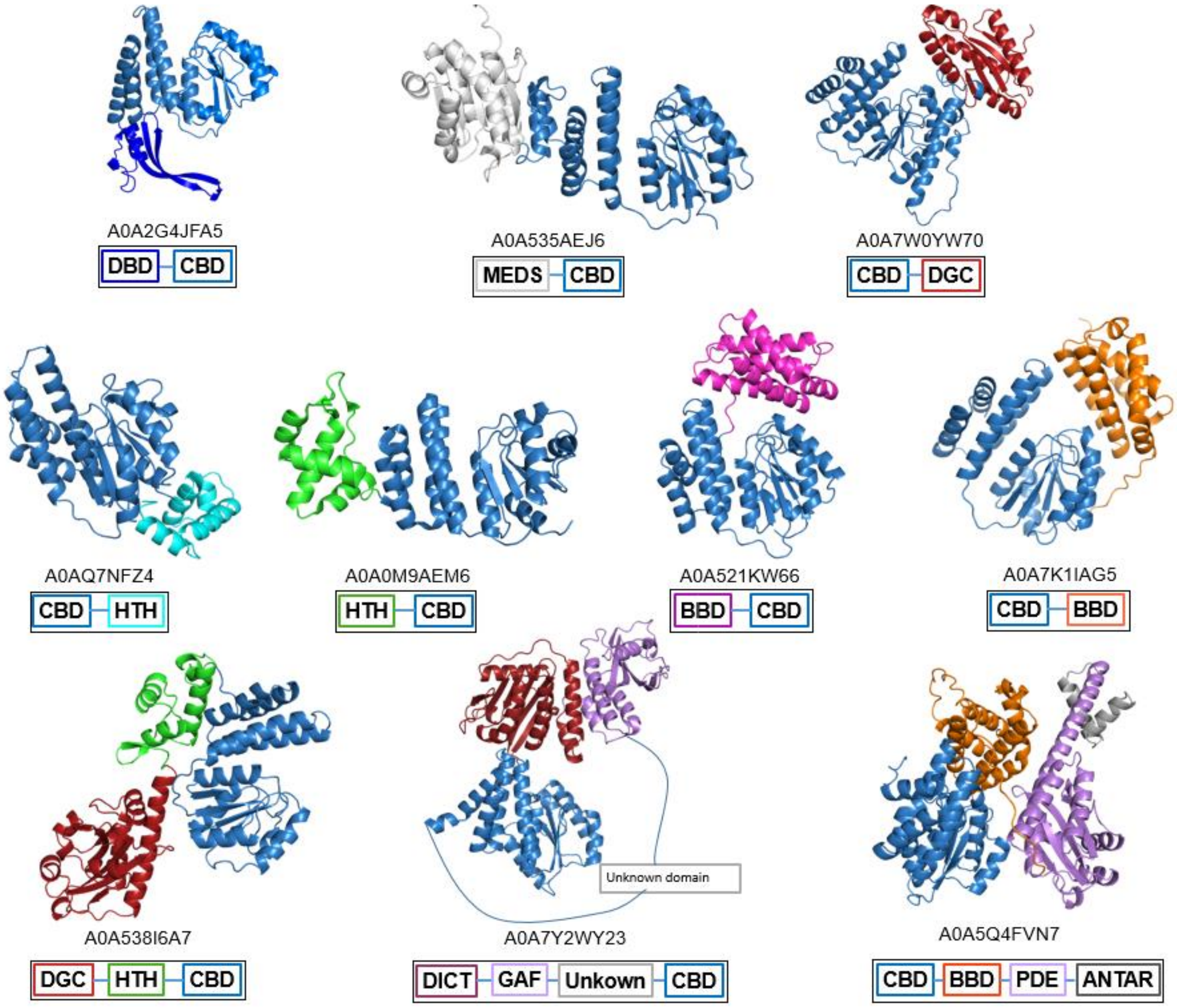

Supplementary Figure S9 - Full length AlphaFold models for representative clusters in sequence similarity networks of putative light-responsive CBD-containing proteins.
